# Visualization of Self-Assembly and Hydration of a β-Hairpin through Integrated Small and Wide-Angle Neutron Scattering

**DOI:** 10.1021/acs.biomac.3c00583

**Published:** 2023-10-24

**Authors:** Harrison Laurent, Matt D. G. Hughes, Martin Walko, David J. Brockwell, Najet Mahmoudi, Tristan G. A. Youngs, Thomas F. Headen, Lorna Dougan

**Affiliations:** † School of Physics and Astronomy, University of Leeds, Leeds, United Kingdom, LS2 9JT; ‡ Astbury Centre for Structural Molecular Biology, University of Leeds, Leeds, United Kingdom LS2 9JT; § School of Chemistry, University of Leeds, Leeds, United Kingdom, LS2 9JT; ∥ ISIS Neutron and Muon Source, Rutherford Appleton Laboratory, Harwell Oxford, Didcot, United Kingdom, OX11 0QX

## Abstract

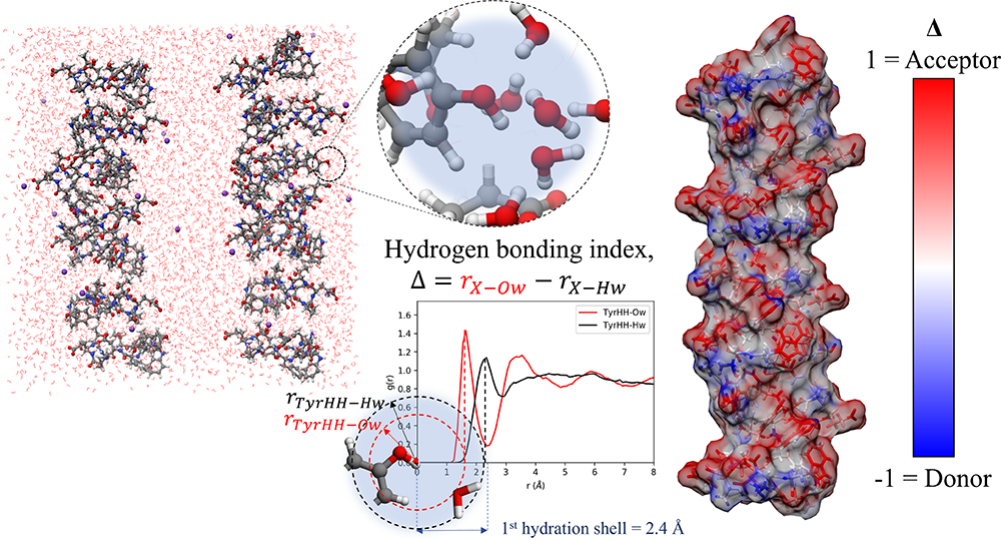

Fundamental understanding of the structure and assembly of nanoscale building blocks is crucial for the development of novel biomaterials with defined architectures and function. However, accessing self-consistent structural information across multiple length scales is challenging. This limits opportunities to exploit atomic scale interactions to achieve emergent macroscale properties. In this work we present an integrative small- and wide-angle neutron scattering approach coupled with computational modeling to reveal the multiscale structure of hierarchically self-assembled β hairpins in aqueous solution across 4 orders of magnitude in length scale from 0.1 Å to 300 nm. Our results demonstrate the power of this self-consistent cross-length scale approach and allows us to model both the large-scale self-assembly and small-scale hairpin hydration of the model β hairpin CLN025. Using this combination of techniques, we map the hydrophobic/hydrophilic character of this model self-assembled biomolecular surface with atomic resolution. These results have important implications for the multiscale investigation of aqueous peptides and proteins, for the prediction of ligand binding and molecular associations for drug design, and for understanding the self-assembly of peptides and proteins for functional biomaterials.

## Introduction

A pervasive challenge across a wide range of scientific disciplines,^1−5^ is understanding how nanoscale properties at the atomic and subatomic length scale result in macroscale observables. Of particular interest to organic and biochemistry is the self-assembly of organic molecules due to their interactions with their aqueous environment.^1,6−9^ To address this challenge, multiscale^1,6,10,11^ and integrative^7,12−14^ approaches are becoming increasingly important, as highlighted by the 2013 Nobel prize in chemistry for coarse grained simulations of protein folding.^15^ Multiscale approaches are often entirely simulation based, from quantum mechanical simulations, through all atom molecular dynamics, to coarse graining approaches.^1,8^ Each method operates at a different spatiotemporal resolution and informs the next scale method, either sequentially,^9^ or in parallel.^16^ Integrative approaches use experimental data from techniques sensitive to nanoscale structure, such as cryo-EM, small-angle X-ray scattering, and nuclear magnetic resonance, to constrain simulation approaches^14^ and employ machine learning (ML)-accelerated computational methods.^17^

**Figure 1:**
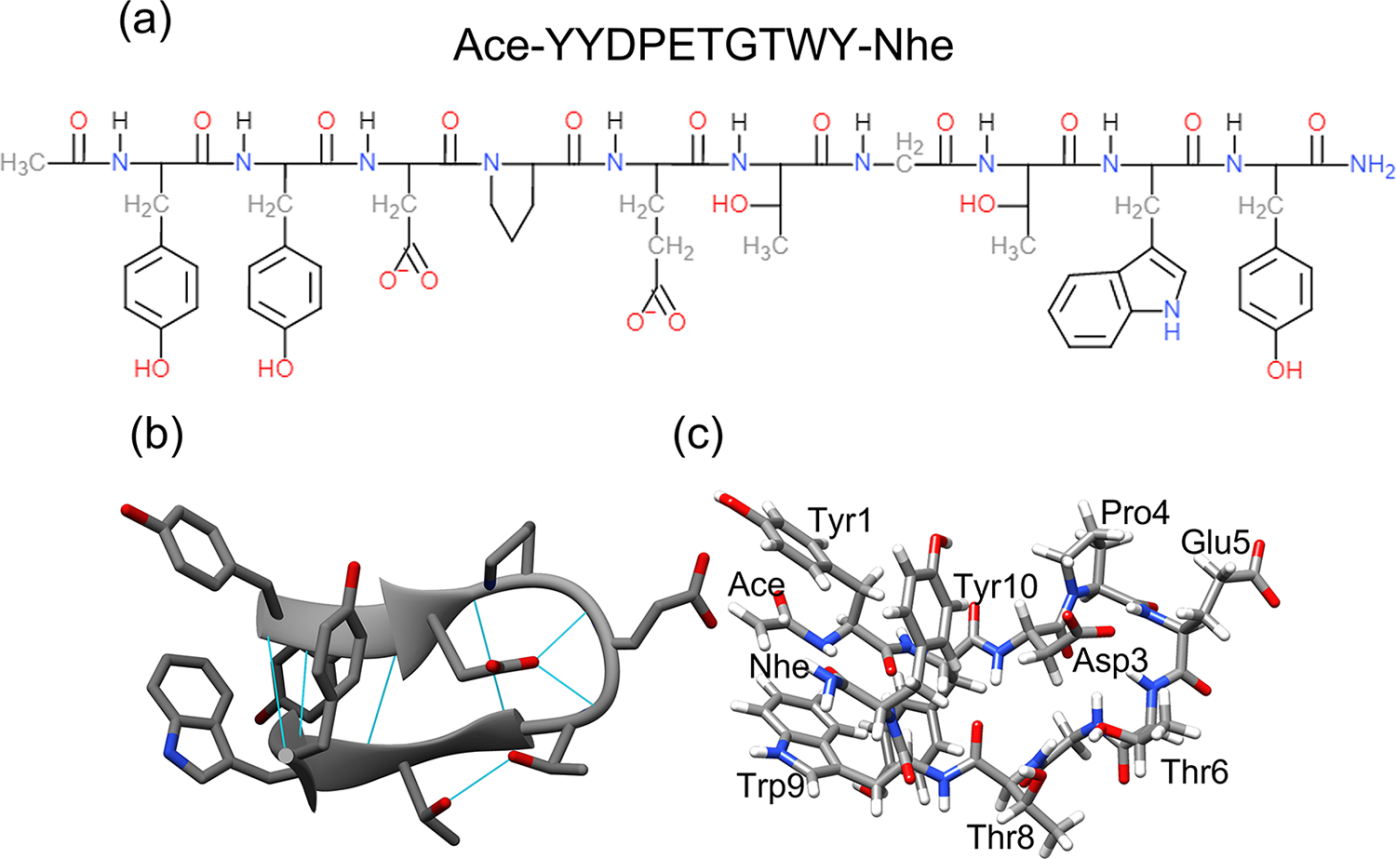
(a) Simplified diagram showing the amino acid sequence of the CLN025 hairpin with capping acetyl (Ace) and amide (Nhe) groups. (b) The folded structure of CLN025 as viewed as a simplified cartoon with intrapeptide hydrogen bonds is shown in turquoise. (c) All atom visualizations viewed from the same orientation as (b) and with visible amino acid side chains and capping groups labeled. Colored according to element (gray = carbon, white = hydrogen, blue = nitrogen, red = oxygen). Amino acids numbered in order of appearance from the N to C terminus. Structure created by manually adding Ace and Nhe caps to crystal structure (PDB code: 5AWL).^19^ Images created using UCSF Chimera.^25^

A particularly powerful experimental technique for integrative multiscale modeling is neutron scattering,^13,18^ as neutrons are deeply penetrating, cause no radiation damage, exhibit much greater sensitivity to hydrogen than X-rays, and allow for deuterium substitution to achieve contrast variation of different chemical environments. In the present work we apply a novel approach of combining small and wide angle neutron scattering (SANS/WANS) measurements to constrain the integrative atomistic modeling technique empirical potential structure refinement (EPSR), yielding an all-atom simulation refined against experimental data of a concentrated aqueous solution of the nanoscale β-hairpin “mini-protein”: CLN025.^19^ CLN025 is a synthetic 10 residue peptide that was chosen due to its well-defined folded structure^19^ and high solubility. The hairpin structure is a variant of a 10 residue synthetic peptide chignolin,^19^ designed using residues 41–56 of protein GB1 as a target scaffold. It shares several key features with naturally occurring proteins, such as it contains only naturally occurring amino acids, it is thermally stable (melting temperature, 69.6 °C), stable reversible folding with a funnel-like energy surface,^20^ and comparable enthalpy of unfolding (47.7 kJ/mol). It is also of considerable interest, as its fast folding time of ∼100 ns,^21,22^ due to a negligible global energy barrier to folding,^22^ means it can be used for all-atom simulations of protein folding^23^ and is rapidly becoming a benchmark for testing new force fields.^24^ Its sequence and structure are shown in Figure 1.

The structure of the CLN025 hairpin derived from X-ray crystallography shows several water molecules hydrating polar residues of the hairpin, suggesting hairpin–water interactions maintain its fold.^19^ It has also been shown that its folding is driven by a gain in water entropy, primarily originating from an excluded-volume effect.^26,27^ Its small size, well-defined structure, and fast folding time make it an ideal model system for all atom simulations of protein folding in explicit water. Such studies have shown that its funnel-like energy landscape is also lost if water is modeled implicitly rather than explicitly,^28^ and is sensitive to the choice of water reference potential/force field in the molecular dynamics simulations,^24,29,30^ with hairpin stability having been shown to be correlated to the average hairpin–water interaction energy.^31^ The hydration of the CLN025 hairpin is therefore of crucial importance to understand its folding behavior.

Given CLN025’s popularity as a tractable model for ultrafast folding and the opportunity it provides for all atom simulations,^24^ it is vital that its hydration structure is accurately understood. Equally important is the understanding of hairpin self-assembly, given that nanoscale β hairpins have become an established building block for the creation of stimuli-responsive biomaterials (hydrogels).^32−34^ A pressing challenge, therefore, is to understand hairpin hydration with atomic resolution in the context of a self-assembled system. This necessitates a multiscale approach. Combining SANS and WANS with computational modeling addresses this challenge. SANS is sensitive to structural correlations involving large scale structures (up to ∼ hundreds of nm) and is well established to study the structure of polymers, hydrogels, and biomaterials.^35−39^ In this work, it allows us to study self-assembly of the CLN025 hairpin in aqueous solution. Conversely, WANS (specifically wide Q-range total scattering) is sensitive to structural correlations involving small scale structures (on the order of ∼ angstroms) and is well established for the study of atomic structure of systems such as aqueous solutions,^40−42^ ionic liquids^43,44^ and crucially to this work, aqueous amino acids.^45−48^ In this work, it allows us to study hairpin hydration with atomic resolution. Multiple length scales are important for the structure of concentrated aqueous peptides, from hydrogen bonds (∼2 Å), through to the monomeric peptide structure (∼10 Å), to large-scale peptide self-assembly (up to ∼100 Å in the case of amyloid fibrils^49^). A combined SANS and WANS investigation is very important for the CLN025 hairpin as it possesses a well-defined folded structure that is defined by its hydration, and it can self-assemble to form larger length scale structures at high concentration. Combining these techniques allows us to map out the hydrogen bonding character of a multiscale self-assembled biomolecular complex with atomic resolution, with important implications for simulation force field design, molecular recognition prediction, and biochemical self-assembly.

## Experimental Section

### Hairpin Synthesis

A microwave-assisted solid-phase Fmoc-based synthesis was completed in a CEM Liberty Blue peptide synthesizer as described previously.^50^ The synthesis was performed at a 0.25 mmol scale on Rink-amide resin using 5 equiv of Fmoc-protected amino acid, 5 equiv of *N*,*N*′-diisopropylcarbodiimide (DIC), and 5 equiv of 2-cyano-2-(hydroxyimino)acetate (Oxyma) in *N*,*N*-dimethylformamide (DMF) with microwave heating at 90 °C. The deprotection was achieved using 20% piperidine in DMF with microwave heating at 90 °C. The procedure was iterated until the complete polypeptide was formed, then the sample was removed, washed through with DMF, resuspended in DMF and reacted for 30 min with 10 equiv of acetic anhydride to form the acetyl cap on the unprotected N-terminus. The hairpin was then cleaved from the resin using 10 mL of a cleaving cocktail containing 92.5% trifluoroacetic acid (TFA), 2.5% water, 2.5% triisopropylsilane (TIPS), and 2.5% 3,6-dioxa-1,8-octanedithiol (DODT). The hairpin therefore differs slightly from the one investigated by Honda et al.^16^ as the hairpin employed in this work has protective acetyl and amide caps at the N and C terminus, respectively. The solution was filtered to remove resin, and diethyl ether was added at −20 °C to precipitate the hairpin. After centrifugation for 5 min at 6000 rpm, the diethyl ether was decanted and the hairpin was rehydrated and freeze-dried. It was later rehydrated and purified using preparative high pressure liquid chromatography (HPLC) coupled with mass spectrometry before being freeze-dried again for storage. High resolution mass spectrometry data and HPLC data, presented in Supporting Information, Figures S1 and S2, confirm that the hairpin was produced with the expected molecular weight and to a high purity. Circular dichroism data presented in Figure S3 demonstrates the secondary structure of the hairpin produced in this work is identical to that produced by Honda et al.,^19^ despite the additional capping groups.

### Resuspension of Hairpin

CLN025 only forms a higher order structure at a high concentration. Consequently, a ratio of 1:500 CLN025:water molecules was chosen, corresponding to 111 mM or 150 mg/mL. Due to the presence of two acidic residues in the hairpin (Glu5 and Asp3), it was dissolved in 250 mM NaOH to aid solubility. This was achieved by packing an Eppendorf with freeze-dried purified hairpin, adding solution, dissolving, and centrifuging at 5000g for 1 min to pellet any aggregated hairpin. The solution was then removed, leaving any visible pelleted hairpin behind and used to dissolve more freeze-dried hairpin packed into a second Eppendorf. This was repeated until the desired concentration was reached, as validated through absorbance spectrometry at 280 nm using an extinction coefficient of 9970 M^–1^ cm^–1^. pH was measured to be 5.5, hence, all amino acids are expected to occur as they would do at neutral pH. Circular dichroism data presented in Supporting Information, Note S1 and Figure S3, suggest that this method of concentrating the hairpin causes negligible irreversible aggregation.

### Neutron Scattering

Neutron scattering relies on the constructive interference of a beam of neutrons diffracted through an experimental sample to extract spatial correlations.^18^ This is monitored according to the wavevector of the scattered neutrons *Q*, which is equal to the difference in momentum between the initial *k*_*i*_ and scattered *k*_*f*_ neutron. In the case of elastic scattering, *Q*, is dependent on the wavelength of the neutron λ and the scattering angle 2θ, as shown in eq 1.
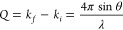
1

The wave vector *Q* is an *inverse* length scale metric. For example, for periodically repeating structures the Bragg condition is satisfied such that the distance between repeated layers, *d*, is inversely related to *Q* as shown in eq 2.
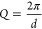
2

Neutron scattering data was collected on the small angle instrument Zoom^51^ at a sample-to-detector distance of 8 m, covering a *Q* range of 0.0022–0.45 Å^–1^ (corresponding to a distance range of ∼14–3000 Å), and the intermediate to wide angle instrument NIMROD,^52^ covering a *Q* range of 0.02–50.0 Å^–1^ (corresponding to a distance range of ∼0.1–300 Å). In both instances, the measured quantity is the differential scattering cross section *I*(*Q*). NIMROD data are conventionally reported in units of barns steradian^–1^ atom^–1^, while Zoom data are reported in units of cm^–1^. The NIMROD data is converted and rescaled to units of cm^–1^ and then stitched to the Zoom data within the software suite MANTID. For SANS analysis, *I*(*Q*) is modeled as proportional to the product of the form factor, *F*(*Q*), that describes the shape of the scattering object and the structure factor, *S*(*Q*), that describes the positional correlations between scattering objects. In WANS measurements, *I*(*Q*) is related by scattering theory to a sum of the partial atomic structure factors *S*_αβ_ that describe positional correlations between distinct atom types α and β weighted by their relative concentrations *c*_*i*_ and coherent scattering lengths *b*_*i*_, as described in eq 3.
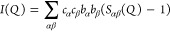
3

Each *S*_αβ_(*Q*) is then related to the associated atom–atom radial distribution function (RDF) by Fourier transform, as shown in eq 4, where ρ is the density in atoms Å^–3^, *g*_αβ_(*r*) is the RDF between atom types α and β, and *r* is the distance in Å. RDFs represent the local density of a given atom type β normalized to the bulk density of β as a function of distance from a given atom type α. Therefore, in the case of liquids, features such as hydration shells occur as a series of broad peaks of diminishing intensity as the local structure around α below *r* ≈ 10 Å gives way to the disordered bulk liquid structure, at which point *g*_αβ_(*r*) = 1.
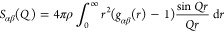
4

### Low *Q* Reduction and Fitting

To aid the following discussion, we will refer to data below *Q* ≈ 0.5 Å^–1^ as “low *Q*”, data around *Q* ≈ 1 Å^–1^ as “mid *Q*”, and data above *Q* ≈ 1 Å^–1^ as “high *Q*”. To allow for fitting and analysis of the mid to low *Q* data, the data for CLN025 suspended in 250 mM NaOH/100% D_2_O solution, from both NIMROD and Zoom, were first buffer subtracted using a 100% D_2_O scan. While it has previously been demonstrated by Imberti et al.^53^ that hydroxide salts have an effect on the structure of water, these results were shown for OH^–^ concentrations in excess of 1:12 mol ratio of salt:water. These concentrations are over 18 times higher than those used in this study, suggesting that the structure of water is insignificantly impacted; hence, pure D_2_O should serve as a reasonable estimate for buffer subtraction. As stated previously, the rescaled NIMROD data were stitched with the absolutely scaled data from the Zoom instrument to extend the low *Q* range by another order of magnitude, from 0.03 Å ^–1^ to 0.003 Å ^–1^. This was fitted with a repulsive elliptical cylinder model with the addition of two Gaussian functions and a power law term shown in eq 5 using SasView (http://www.sasview.org). The addition of the Gaussian functions is to accurately model the mid-*Q* data, while the power function is to capture the increasing intensity profile at the lowest *Q* values.
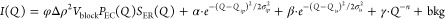
5where φ, Δρ, and *V*_block_ are the volume fraction, neutron contrast, and volume of the CLN025 stack scattering object, respectively, *P*_EC_(*Q*) is an elliptical cylinder^54^ form factor and *S*_ER_(*Q*) is a electrostatic repulsion structure factor.^55,56^ α and β are scaling factors to describe the amplitude of the two Gaussian functions centered around *Q*_*ip*_ and *Q*_*is*_, respectively, observed in the mid-*Q* data. γ is the scaling factor for the power law described using exponent *n* observed in the lowest *Q* data (*Q* < ∼ 10^–2^ Å^–1^). The background, bkg, is a constant that models the incoherent background scattering signal.

### Structural Refinement

The experimental WANS data from NIMROD was first corrected for incoherent, multiple scattering, and attenuation effects using Gudrun software.^57^ This data was analyzed using the Monte Carlo based structural refinement technique EPSR^58^ over the range 0.05 < *Q* < 30 Å^–1^. This technique addresses the challenge that for a system containing *J* distinct atomic species, the number of distinct interatomic correlations *N* is equal to *J*(*J* + 1)/2, hence, deconvolution of total scattering data arising from the weighted contribution of every distinct interatomic correlation present within the sample into a complete set of partial structure factors by studying *N* distinct isotopic variants rapidly becomes impossible. EPSR therefore provides atomistic information to produce a complete set of partial structure factors by building a simulation that is simultaneously constrained by experimental data on fewer distinct isotopic variants (typically between 3 and 7).^58^ Neutron scattering combined with EPSR has previously been well applied to the study of simple binary solutions, including those containing biomolecule building blocks such as amino acids^45,47,48^ and short peptides,^59−61^ as well as a host of other aqueous systems including pure water under various pressures and temperatures,^40,62^ alcohols,^63−65^ salts,^53,66−68^ and other osmolytes.^69−72^ EPSR builds a simulated box of molecules matching the concentrations and density of the experimental sample, with each atom characterized by a reference potential consisting of a charge *q* and the Lennard–Jones (LJ) parameters σ and ε using the standard Lorentz–Berthelot mixing rules. Exchangeable hydrogens are accounted for when calculating the predicted weights of each interatomic correlation to the total scattering data for each isotopic variant. The simulation is then equilibrated through a Monte Carlo procedure using this reference potential. Once this is complete, an iterative, evolving empirical potential is applied, derived from the difference between the predicted scattering data from the simulation and the experimentally obtained scattering data. The simulation continues until a satisfactory agreement exists between the simulation and the supplied scattering data. Monte Carlo steps are allowed to continue further for >1000 iterations to gather statistics on interatomic correlations. The dynamic liquid nature of the sample requires this as atomic positions will be constantly evolving, and a single “crystal-like” structure with well-defined atomic positions is unfeasible. Gathering statistics over large numbers of iterations therefore allows one to determine average interatomic correlations and positional uncertainties through tools such as RDFs. The result is an ensemble of structures from a simulated box of atoms whose predicted scattering data is consistent with the measured scattering data. The nature of this procedure does not guarantee a single unique solution to the supplied scattering data, but it is consistent with the scattering data and based on sensible starting potentials and known intramolecular structures. The workflow of these procedures and quantification of quality of fit is outlined in Supporting Information, Figure S4.

The EPSR simulation contained 6944 water molecules, 14 CLN025 molecules, and 28 Na^+^ ions (0.1 atoms/Å^3^). The concentration of Na^+^ ions is slightly lower than that in the experimental sample. This is a simple way to ensure charge neutrality without requiring free hydroxide ions, as there is no capacity within EPSR for dissociation/reassociation of water into its constituent ions (free hydrogen, hydroxide, hydronium, etc.^73^). The reference potential for water was the SPC/E model,^74^ the Na^+^ force field was taken from Mancinelli^68^ and the CLN025 force field from the AMBER-FB15 potential from McKiernan et al.^23^ The number of atom types for CLN025 was reduced by grouping similar atoms of a similar charge as described in Supporting Information, Note S2. The raw and modified force fields can be found in Supporting Information, Tables S1 and S2.

The CLN025 hairpin was modeled within EPSR by directly incorporating its crystal structure as reported by Honda et al.^19^ and multiple hairpin self-assembly was incorporated based on results from the SANS analysis. The CD data reported in Supporting Information, Figure S3 show that this is a reasonable approach, as the strongly overlapping spectra suggest essentially identical secondary structure between the hairpin used in this work and that of Honda et al. The acetyl and amide caps were then added to the N and C termini, respectively, without further structural minimization. This method was chosen as the minimization step employed through EPSR, which causes the hairpin bonds along the length of the hairpin backbone to be distorted away from the *trans*-conformation and, therefore, produces a physically unreasonable structure. No bond rotations were permitted, and the CLN025 molecule therefore remains folded during the simulation by virtue of intramolecular harmonic potentials between 1–2 neighbors (bonded atoms), 1–3 neighbors (bond angles), and 1–4 neighbors (dihedral angles). These are defined by an average distance and a width term that allows for natural fluctuation of bond lengths/angles and mimics thermal fluctuations.^75^

## Results and Discussion

### SANS Analysis–Hairpin Self-Assembly

**Figure 2:**
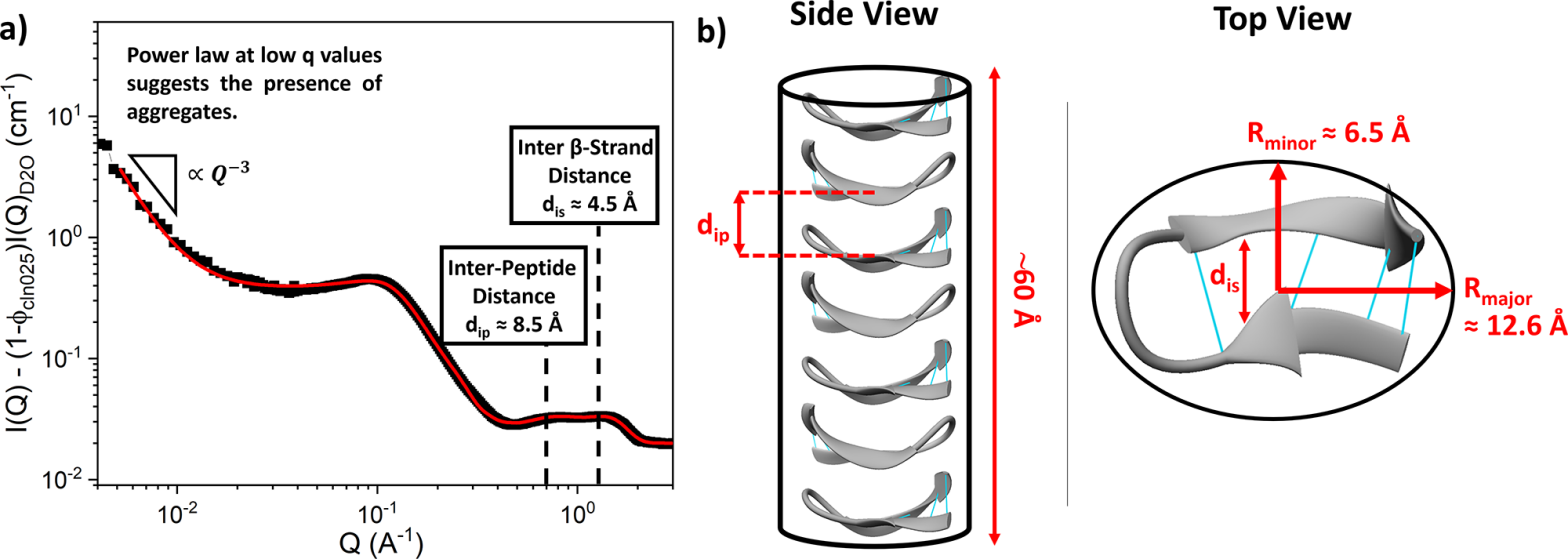
(a) Low *Q* data of CLN025 hairpin resuspended to 150 mg/mL in 100% D_2_O after background subtraction of the D_2_O signal (see Experimental Section), fitted using a repulsive elliptical cylinder model^54^ with the addition of two Gaussian peak functions (eq 3, red line). (b) Schematic showing the association of seven CLN025 in solution at 150 mg/mL, forming an elliptical cylinder of length ∼60 Å and cross-section aspect ratio of ∼1.94. CLN025 intrapeptide hydrogen bonds are shown in turquoise.

Figure 2a shows the intensity profile of CLN025 at 150 mg/mL in D_2_O (with 250 mM NaOH) from the combined neutron data obtained on both the NIMROD and Zoom instruments. Below *Q* = 0.03 Å^–1^ a power law with decreasing *Q* is observed, yielding a fitted exponent of 3 ± 0.2, which suggests the presence of large (>6000 Å) dense fractal aggregates of unfolded protein. However, this power law is not observed over the entire *Q* range, suggesting that the volume fraction of these large aggregates is low. CD analysis does not show the presence of any aggregates, confirming that any aggregates present, while large, must be at relatively low concentrations compared to the remainder of the CLN025 hairpin, as detailed in Supporting Information, Note S1. The intensity profile in the region 0.03 < *Q* < 3 Å^–1^ is characterized by two overlapping peaks at ∼1.0 Å^–1^, and a power law increase in the intensity ∝ *Q*^–2.5^ in the region of 0.1 < *Q* < 0.5 Å^–1^ followed by an inflection at ∼0.1 Å^–1^. The overlapping peaks at ∼1.0 Å^–1^ suggest the presence of repeated characteristic structural length scales, while the *Q*^–2.5^ power law suggests there is a nonspherical 3D scattering object, e.g., a thick rod and the inflection at *Q* ∼ 0.1 Å^–1^ suggests that these objects are electrostatically repulsive. This is supported by previous observations of electrostatic repulsion between proteins in aqueous solution measured by SAXS at ionic strengths <1 M^76^ (ionic strength of NaOH buffer used in this work is 250 mM, however neutralization of the OH^–^ ions by acidic residues on CLN025 will yield a further decrease in ionic strength).

Fitting eq 5 to the data in Figure 2a (see Supporting Information, Note S3 for further details) suggests that CLN025 hairpins assemble into a cylinder with a length of 60 ± 10 Å and an elliptical cross section with *R*_minor_ and *R*_major_ of 6.5 ± 0.6 and 12.6 ± 0.8 Å, respectively (Figure 2b). The fitted cross-sectional radii match well with the polar (11 Å) and major equatorial (6.6 Å) radii of the hairpin (Supporting Information, Figure S7), suggesting that the plane of the β-hairpin CLN025 is aligned perpendicular to the axis of the elliptical cylinder. If the hairpin is aligned such that the self-assembled cylindrical structure is made up of a planar stacked hairpin, then we would expect there to be 7 hairpins per cylinder on average, as the minor equatorial diameter of the hairpin is 9.6 Å (Supporting Information, Figure S7). As the average charge of CLN025 at neutral pH is −2 e, we would expect the average overall charge of the self-assembled cylinder to be −14 e; indeed, from the fitting (Figure 2a) we extract an estimate of the total charge of the self-assembled cylinder to be −14 ± 1 e. Furthermore, the fitted peaks suggest that there are two repeated characteristic lengths in the self-assembled CLN025 cylinder, 8.5 ± 0.5 and 4.5 ± 0.6 Å, we speculate that these characteristic lengths correspond to the inter-hairpin distance, *d*_*ip*_, and the inter-β-strand distance, *d*_*is*_, respectively (Figure 2b). Overall, the *Q* data between 0.003 Å^–1^ and 3 Å^–1^ suggest that there is association between the CLN025 hairpins that self-assemble into a cylinder of planar stacked hairpins. Similar stacked cylindrical structures of β-sheet peptides have been observed in other experimental systems including amyloid fibrils and peptide-based hydrogels.^32,33,77^ It should be noted that in such systems the axis of the fibril is orthogonal to the plane of the biomolecules, whereas for CLN025 it is parallel. Each term within eq 5 was fit individually to the scattering data to ensure stability effects of simultaneously fitting multiple parameters were avoided, as detailed in SI (Supporting Information, Figure S8). The χ^2^ values of this procedure are also reported in the SI.

### WANS Analysis–Hairpin Hydration

**Figure 3:**
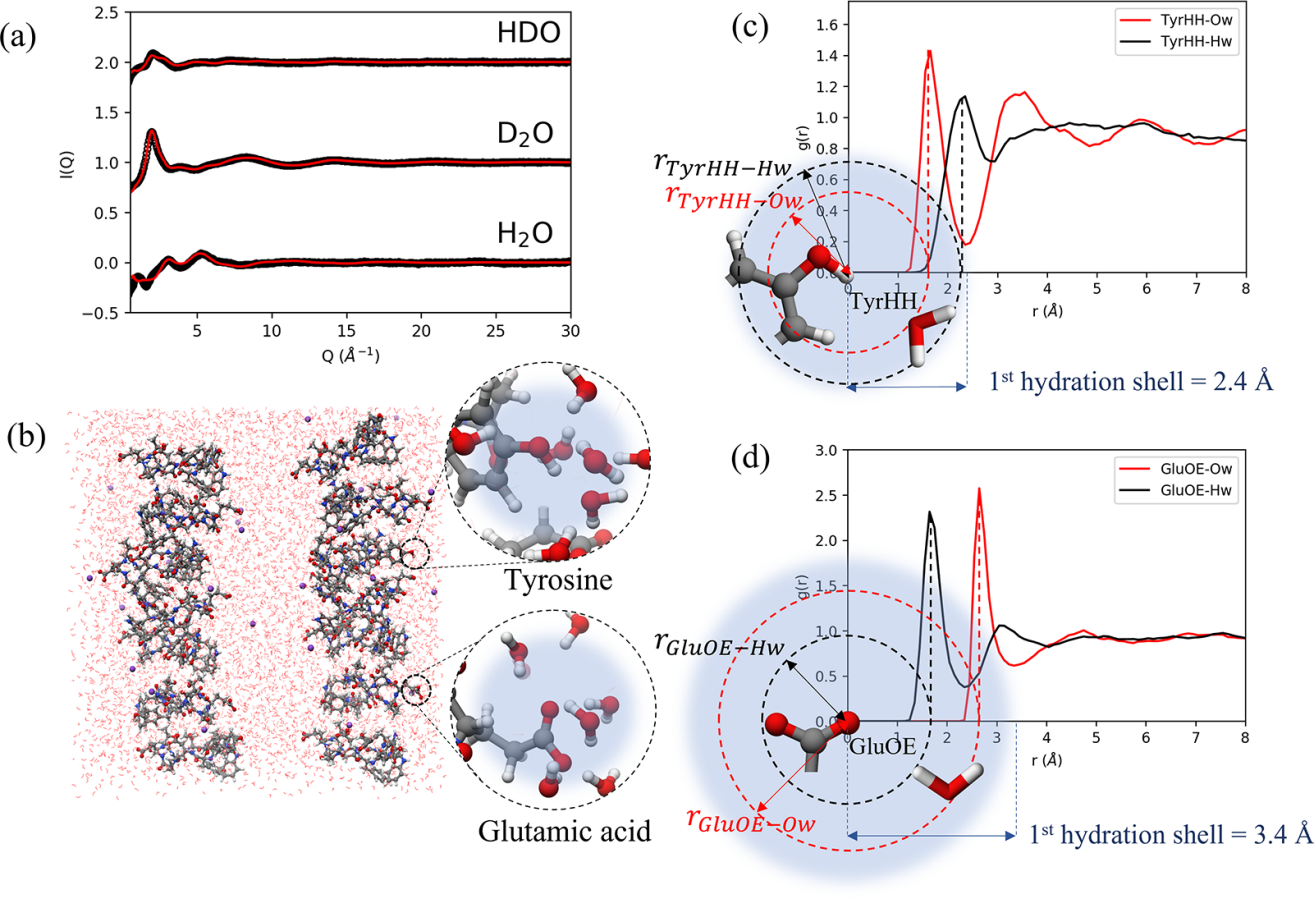
(a) Differential scattering cross section (*I*(*Q*)) obtained from the neutron diffraction data on the NIMROD instrument (black circles) for the CLN025 hairpin in H_2_O, D_2_O, and an equimolar mixture of H_2_O and D_2_O (HDO) at 298 K and the EPSR simulated data (red line). Units of the differential cross sections are barns/atom/sr (1 barn = 10^–28^ m^2^). (b) Simulation box produced by EPSR with CLN025 stacks shown as ball and sticks, Na^+^ ions shown as balls, and water molecules shown as wires. Zoomed areas demonstrate hydration around the Tyr group and Glu side chains. Elements colored according to gray = carbon, red = oxygen, white = hydrogen, blue = nitrogen, purple = sodium. (c) Radial distribution function of water oxygen (Ow) and water hydrogen (Hw) around positively charged tyrosine hydroxyl hydrogen (TyrHH). Water molecules are shown to orient hydrogens away from the TyrHH, resulting in a difference in peak positions between the TyrHH-Ow and TyrHH-Hw peaks of −0.63 Å. (d) Radial distribution function of water oxygen (Ow) and water hydrogen (Hw) around negatively charged Glu side chain oxygen (GluOE). Water molecules are shown to orient hydrogen atoms toward from the GluOE, resulting in a difference in peak positions between the GluOE-Ow and GluOE-Hw peaks of 0.95 Å.

The SANS data suggest a stacked hairpin structure involving seven CLN025 monomers; however, it is incapable of providing the structure of a stack with atomic resolution. To achieve this length scale resolution, we built a stack of seven all atom hairpin monomers using a custom built molecular docking Monte Carlo simulation (Supporting Information, Note S4 and Figures S10–S15). Following the building of the 7-hairpin stack, two stacks were loaded into EPSR and, hence, a total of 14 hairpin monomers, into a cubic box of dimension 61.506 Å separated by a horizontal distance of half the box width. This represents a necessary simplification of the modeled system, as in the solution we expect from the 2D SANS pattern (Supporting Information, Figure S9) that the stacks will be randomly oriented with respect to each other and not fixed in their positions. However, the primary objective of the EPSR analysis is to understand local hydration, which should not be affected by the interstack structure at longer length scales. Each CLN025 molecule was tethered to its starting coordinate by its center of mass to preserve the stack; however, other movements (molecule rotations, bond stretching and bending, etc.) were permitted. The hairpin stacks were then hydrated using 6944 water molecules and 28 Na^+^ ions to ensure overall charge neutrality leading to a density of 0.1 atoms/Å^3^. Following the procedure of EPSR, the box was allowed to equilibrate under a reference potential and then the empirical potential. Statistics were then gathered over >3000 iterations. The final fits between experimental data and EPSR simulation are shown in Figure 3a, and the quality is given by *R* ∼ 0.01, as defined in Supporting Informatin, Figure S4. The fits are observed to be of high quality above *Q* ∼ 1 Å^–1^, however, the strong upturn at low *Q* is only observed in the fully H_2_O sample, as shown in Supporting Informatin, Figure S16. This suggests that treating the hairpin self-assembly as two stacks of 7 hairpins held at fixed positions partially captures the low *Q* behavior. In the future, a larger box containing multiple hairpin stacks that are free to diffuse may be required to fully capture the low *Q* behavior. It is also important to note that the simulated scattering data produced through EPSR is only reliable to a minimum *Q* of 4π/*L*_box_, where *L*_box_ is the full width of the cubic simulation box, hence, a larger box will intrinsically improve the ability of EPSR to fit the low *Q* behavior. However, this is outside the scope of the EPSR’s current capabilities. The data above *Q* ∼ 1 Å^–1^ contains information on small scale structures, such as peptide–water and water–water interactions and, hence, can be reliably studied to inform hairpin hydration characteristics.

**Figure 4:**
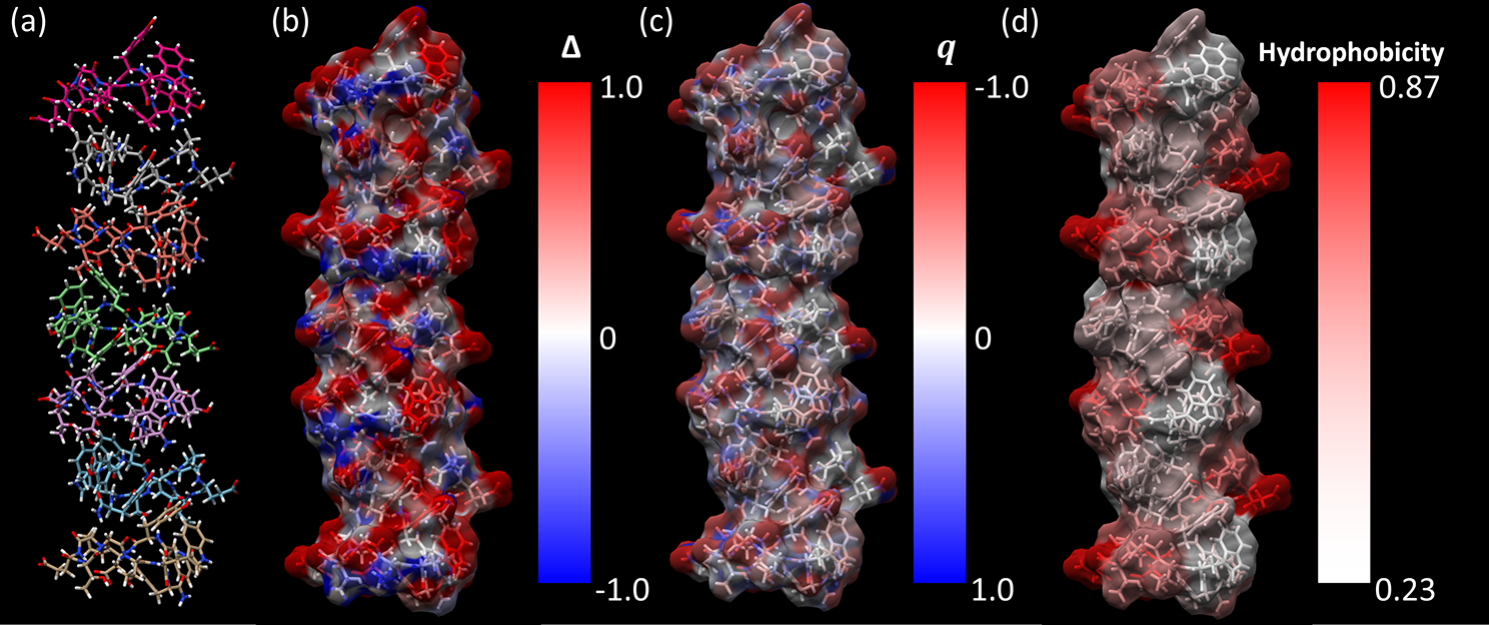
(a) All atom views of an assembly of seven hairpins as viewed from the front. Carbon atoms on each hairpin colored differently to aid in distinction between neighboring hairpins. Other atom species colored according to oxygen, red; nitrogen, blue; hydrogen, white. (b) Calculated Δ values for each atom on the hairpin surface colored from red to blue as Δ varies from 1 to −1 as viewed from the front. (c) Charge of each atom as described in Supporting Information, Table S2. (d) Normalized hydrophobicity of each amino acid species where 1 is hydrophilic as 0 is hydrophobic. Hydrophobicity gathered by normalizing values found in previously published hydrophobicity indices.^78−82^

Using the accumulated data from the >3000 iterations it is possible to derive the hydration structure of each atom belonging to the peptide stacks. A snapshot of a single iteration for the hydration structure around a tyrosine side chain and a glutamic acid side chain is shown in Figure 3b. This is quantified over the >3000 iterations by considering the site–site RDFs between the hairpin atoms and the hydrating water molecules as described in the experimental details. The RDFs for water around a tyrosine side chain hydroxyl hydrogen TyrHH and a glutamic acid side chain oxygen GluOE are shown in Figure 3c,d. Here it can be observed that the hydrating water molecules tend to orient their positively charged hydrogens away from a positively charged hydrogen bond donating atom, such as TyrHH, or orient a single positively charged hydrogen toward a negatively charged hydrogen bond accepting atom, such as GluOE. This observation allows the hydrogen bonding characteristics of the hairpin surface to be described. The first peak in the XH_w_ RDF *r*_X-H_w__ will therefore occur at a shorter distance than the first peak in the XO_w_ RDF *r*_X-O_w__ for a hairpin hydrogen bond accepting site X, as shown in Figure 3d for GluOE and *vice versa*, as shown in Figure 3c for TyrHH. For hydrogen bond accepting sites, the difference between these two peaks was measured (*r*_X-O_w__ – *r*_X-H_w__) and normalized to the OH bond length (*R*_donor_ = 0.96 Å) to yield the hydrogen bonding index Δ. The Δ therefore ranges from 0 to 1 for hydrogen bond accepting sites where 1 is a perfect hydrogen bond acceptor and 0 is a site that causes the hydrating water molecule to show no tendency to orient its hydrogen atoms toward or away from the site. For hydrogen bond donating sites *r*_X-O_w__ – *r*_X-H_w__ was normalized to *R*_acceptor_, as detailed in eq 6.
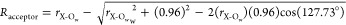
6*R*_acceptor_ represents the *r*_X-O_w__ – *r*_X-H_w__ distance that would occur if the hydrating water molecule were to orient both hydrogens simultaneously as far away as possible from site X, to yield the value Δ. The Δ ranges from 0 to −1 for hydrogen bond donating sites, where −1 represents a perfect hydrogen bond donor and 0 again is a site that causes the hydrating water molecule to orient its hydrogens toward or away from the site. Δ values calculated for each atom on the hairpin stack surface with identical atoms on each of the 14 hairpins are assumed to have identical Δ values. The results of this procedure are shown in Figure 4a,b.

### Hydrogen Bonding and Hydrophobicity

We present a new integrative experimental approach that simultaneously gives structural insight into hydrogen bonding and hydrophobicity of a self-assembled biomolecular surface at atomic resolution. This powerful approach reveals important insight into the specific hydration characteristics of the biomolecular surface. For example, in Figure 4 we consider the hydrogen bonding index Δ (Figure 4b) alongside the charge of each atom as described in Supporting Information, Table S2 (Figure 4c) and normalized values for amino acid hydrophobicity available in the literature^78−82^ (Figure 4d). By comparing Figure 4b,d, we observe that considering each atom individually not only gives a much higher spatial resolution regarding hydration than a single hydrophobicity value for a complete residue, but also allows us to distinguish between hydrogen bond donors/acceptors. This distinction is important for predicting molecular assembly in solution, as a hydrogen bond donating residue would have an identical hydrophobicity value to an equally effective hydrogen bond accepting residue, even though they could interact in opposite manners with a hydrogen bond forming solute. A more detailed comparison between hydrophobicity determined through the work herein and published hydrophobicity scales is presented in Supporting Information, Note S5 and Figure S17.

As expected, strongly negatively charged atoms are likely to act as hydrogen bond acceptors and therefore have positive Δ values and *vice versa*. Comparing Figure 4b to c shows that this is indeed largely true; however, an important observation is that strongly charged solvent exposed areas according to the EPSR reference potential (Glu side chain oxygens, Tyr side chain hydroxyl groups, etc.) confer hydrogen bonding character to their surrounding areas. For example, it can be observed that Trp, despite consisting largely of essentially neutrally charged atoms, obtains a strong hydrogen bond accepting character (positive Δ). This may be due to the stacking of the aromatic rings between the Trp side chain and 3 neighboring Tyr side chains resulting in electron delocalization. It can also be observed that Pro obtains hydrogen bond donating character (negative Δ) due to its position between several neighboring strong hydrogen bond accepting sites. The reported data therefore suggest that the hydrophobicity or hydrogen bonding character of an amino acid depends strongly on its immediate chemical environment and the relative orientation of its backbone/side chains.

Surface hydration characteristics have also been shown to be dependent on the solvent environment^73^ via the effects of physical parameters such as temperature,^83,84^ pressure,^85,86^ and chemical parameters, such as the presence of solutes, such as salts,^87,88^ and osmolytes, such as TMAO^89,90^ and urea.^91,92^ EPSR has already proved useful in this respect by studying interactions between small biologically relevant osmolytes and small model biomolecules such as amino acids^48^ and short peptides.^59,60^ By employing an integrated SANS and WANS approach, we have achieved a step change in complexity, focusing on a concentrated hairpin solution, which self-assembles into a high aspect ratio macrostructure with a large surface area with varied chemical and topographical character. As the Dissolve platform continues to be developed,^93^ there will likely be future opportunities to incorporate larger box sizes with integrated molecular dynamic simulations. The model system and detailed analysis presented here provide an ideal model system for structural and dynamic investigations into complex multiscale biomolecular systems.

## Conclusions

In this work we have used SANS data to constrain the computational modeling technique EPSR to analyze WANS data of a concentrated aqueous hairpin with atomic resolution. Through this methodology we have been able to provide detailed analysis on the hydrogen bonding and hydrophobic character of the self-assembled hairpin superstructure surface. This integrated SANS, WANS, and EPSR approach offers enormous potential to the study of biomaterials, such as peptide hydrogels. These exciting materials rely on the self-assembly of short peptides (∼2–20 amino acids) to form soft materials that retain large volumes (>90%) of water with tunable physical properties.^34,94−97^ Their biocompatibility makes them well suited for a range of applications, including cell culturing,^34,98^ drug delivery,^99,100^ bone regeneration,^101^ and energy transport.^102^ The self-assembly of such materials is strongly dependent on the solvent environment, commonly controlled through pH,^98−101^ and the properties of the peptide building block, such as hydrophobicity.^95,98,101^ The techniques presented herein could therefore provide valuable atomic scale insight into the large scale self-assembled structures and the small-scale hydration/molecular association structures present in these materials to facilitate their rational design.

We envisage that this could occur in several different ways. As the current limitation of EPSR is approximately 140000 atoms^93^ (simulation size in this work was 23268 atoms), atomistic resolution of a complete hydrogel network on the order of 100s of nm would be unreachable, however more focused simulations of hydration around individual structural motifs of interest, such as single fibers, branches, or entanglements that have been previously observed in peptide hydrogels,^33^ could potentially be achieved. With the development of the Dissolve software,^93^ access to potential refinement techniques on larger simulation sizes ca. 10^6^ atoms is now possible. Coupled with the ability to employ directed construction of simulation starting points, permitting bias toward specific structural motifs and arrangements as the starting point for refinements, Dissolve has the potential to open up structural analysis of these larger and more complex systems. This could allow for the study of a small model hydrogel featuring several structural motifs to be present within the same simulation. In addition, further developments of the coarse grained simulation scattering calculation tool MuSSIC would allow for neutron diffraction constrained computational modeling of yet larger and more complex systems.^103^

Finally, we highlight the importance of the results presented herein to the field of biomolecular simulation. An atomistic mapping of the surface hydrophobicity of a peptide or protein is an important consideration for biomolecular engineering and modeling,^104^ such as in the coarse-grained MARTINI,^105^ and PRIME models.^106^ The MARTINI model has been primarily applied to the study of peptide assembly,^107,108^ proteins in lipid membranes,^109,110^ and protein conformational changes,^111,112^ whereas PRIME is mainly applied to the study of protein aggregation.^113,114^ In both instances, side chains are described using coarse-grained beads that explicitly describe hydrogen bonding and hydrophobicity. The information gathered through the study herein can help parametrize such force fields, for example the description of hydrogen bonding used in the MARTINI force field, in which coarse grained sites are described by hydrogen bonding ability (donor, acceptor, both, none) and polarity (1–5). The site-specific RDFs generated through this approach can also aid in the development of force fields through processes, such as iterative Boltzmann inversion,^115^ inverse Monte Carlo,^116^ or Newton inversion.^117^

## Data Availability

All raw data used to prepare the following work can be found at 10.5518/1345. Raw neutron scattering data on NIMROD instrument at ISIS facility can be found at 10.5286/ISIS.E.RB2010648. Raw neutron scattering data on Zoom instrument at ISIS facility can be found at 10.5286/ISIS.E.RB2220774

## Supporting Information Available

Explanations of csv file formats and python scripts, used to generate data presented. CLN025 characterization data through HRMS, HPLC, and CD. Force field parameters, atom naming conventions, and workflow used in EPSR analysis. Equations and fits to SANS data. Elliptical cross sections predicted for CLN025 monomer using UCSF Chimera. Energy maps for rigid body docking simulations used to optimize hairpin stacks. EPSR fits to WANS data. Conversion of Δ data to a full amino acid hydrophobicity score for comparison with previous literature (PDF)
