# Supplementary material for: Visualization of Self-Assembly and Hydration of a β-Hairpin through Integrated Small and Wide-Angle Neutron Scattering
Source: Biomacromolecules. 2023 Oct 24;24(11):4869–79. doi: 10.1021/acs.biomac.3c00583 (PMC10646990; doi:10.1021/acs.biomac.3c00583)
Supplement: Supplementary file 1 — bm3c00583_si_001.pdf [file bm3c00583_si_001.pdf]

Supplementary Information

Visualisation of self-assembly and hydration of a β-hairpin through integrated small and wide-angle neutron scattering

Harrison Laurent, Matt D.G. Hughes, Martin Walko, David J Brockwell, Najet Mahmoudi, Tristan G. A. Youngs, Thomas F. Headen, Lorna Dougan*

1. Supplementary Data

EPSR results 05012023.csv – contains the raw data used to produce the hydrogen bonding index Δ data presented in figure 5(b) and 5(c). File organised as such:

- Column 1 "Atom number": number corresponding to each atom that occurs in a CLN025 monomer
- Column 2 "AA": amino acid to which the atom belongs within CLN025. Amino acids named according using standard three letter shorthand. Ace and Nhe correspond to acetyl and amide caps respectively. In cases where amino acid species occur more than once in CLN025 (Tyr and Thr) an additional number is given which distinguishes the amino acid. Numbered in order in which they occur reading from the N to C terminus
- Column 3 "Element": atom element
- Column 4 "Atom type": Atom type specifier as they belong to each amino acid as described in figure S1
- Column 5 "Chimera name": atom name as they occur in the visualisation software "Chimera" which was used to produce figures involving CLN025 structures/self assembly
- Column 6 "Solvent exposed?": Specifies whether the atom is solvent exposed in the CLN025 monomer. N corresponds to not solvent exposed and Y corresponds to solvent exposed
- Column 7 "Charge": charge of atom as specified by the EPSR reference potential given in Table S2 given in units of the elementary charge *e*
- Column 8 "Ow peak": position of the first peak in the atom – water oxygen RDF given in units of Å
- Column 9 "Hw peak": position of the first peak in the atom – water hydrogen RDF given in units of Å
- Column 10 "Ow – Hw" : difference between the position of the first peak in the atom – water oxygen RDF and the atom – water hydrogen RDF
- Column 11 "pos scaling": dividing positive values in column 10 by the OH bond length in water taken to be 0.96 Å such that positive values now lie between 0 and 1
- Column 12 "perfect HB acc Hw": calculating the position the first peak in the atom – water hydrogen RDF would occur if the hydrating water molecules are behaving as ideal hydrogen bond acceptor (orienting both hydrogens as far away from the CLN025 atom as possible)
- Column 13 "perfect HB acc Ow – Hw": difference between the position of the first peak in the atom – water oxygen RDF and the calculated position of the first peak in the atom – water hydrogen RDF if the hydrating water molecule is behaving as an ideal hydrogen bond acceptor
- Column 14 "neg scaling": normalising negative values calculated in column 10 by the values calculated in column 13 such that negative values now lie between 0 and 1
- Column 15 "Final list": list of normalised values calculated in columns 11 and 14 that retains whether the unnormalized value was positive or negative such that all values now lie between -1 and 1

Calculated RDFs between CLN025 atoms and hydrating water molecules. File naming convention is "CLN025_X_water_Y.rdfdep1_2" where X corresponds to the CLN025 atom number as specified in "EPSR results 05012023.csv" and Y corresponds to the water molecule atom number, where 1 corresponds to oxygen and 2 corresponds to hydrogen

2. Supplementary Python Scripts

CLN025 dimer optimisation.py: python script designed to find the most energetically favourable conformations between a "host" CLN025 molecule and a "docking" CLN025 molecule separated by a distance of 8.5 Å normal to the plane of the two hairpins. Script operates by the following procedure:

- Reads in .xyz/.ato files describing CLN025 molecules. When optimising the positions of the first pair of CLN025 molecules (dubbed positions 0 and 1), only a single CLN025 file is required as the CLN025 position 0 is manually defined as described below as "CLN025_flat". When building subsequent dimers the optimised conformation of CLN025 position 1 is used as the host molecule and a new CLN025 molecule CLN025 position 2 is defined. This process is iterated until a stack of seven monomers is created
- Creates arrays defining the Lennard Jones parameters and charge associated with each atom in the CLN025 molecule as defined in table S2
- Defines function "move_molecule", which allows for translations in the x, y and z directions, as well as rotations of the CLN025 molecule around the origin according to the Euler angles α, β and γ
- Defines function "E_calc", which calculates the sum of the interaction energy using the Lennard Jones and charge parameters between each pair of atoms in the two CLN025 molecules
- Creates an initial conformation for a CLN025 molecule "CLN025_flat", which minimizes z coordinate for each atom in a CLN025 monomer such that the full hairpin lies as flat in the xy plane as possible
- Defines Monte Carlo simulation functions "optimise_trans" and "optimise_rot" which allow Monte Carlo simulations to be performed between two CLN025 molecules through translations and rotations respectively
- Performs docking simulation by positioning a host CLN025 molecule with its centre of mass at the origin and a docking CLN025_flat molecule with its centre of mass 8.5 Å above the host molecule in the z direction. The docking molecule is then moved in 0.5 Å increments between -8 and 8 Å in the x direction and between -5 and 5 Å in the y direction and allowed to perform 100 Monte Carlo rotation steps by an angle 1/280 radians at each location. The lowest calculated interaction energy is recorded at each xy coordinate
- Results plotted in a contour graph. The 5 contour graphs used to construct the 7 membered CLN025 stack are shown in figures S5-9
- Once lowest interaction energy position in xy is determined the simulation is allowed to optimise further at this position by performing 1000 Monte Carlo rotation steps
- Optimised docking conformation is written to a .xyz file

3. Supplementary Tables, Figures and Notes

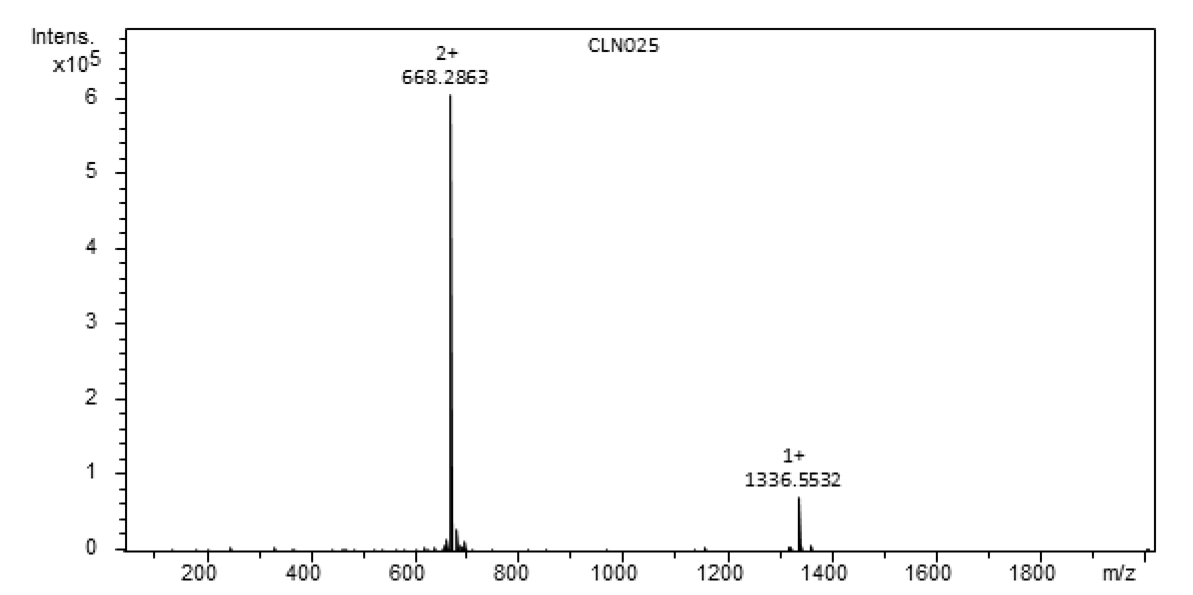

Figure S1. High resolution mass spectrometry data of purified CLN025 peptide

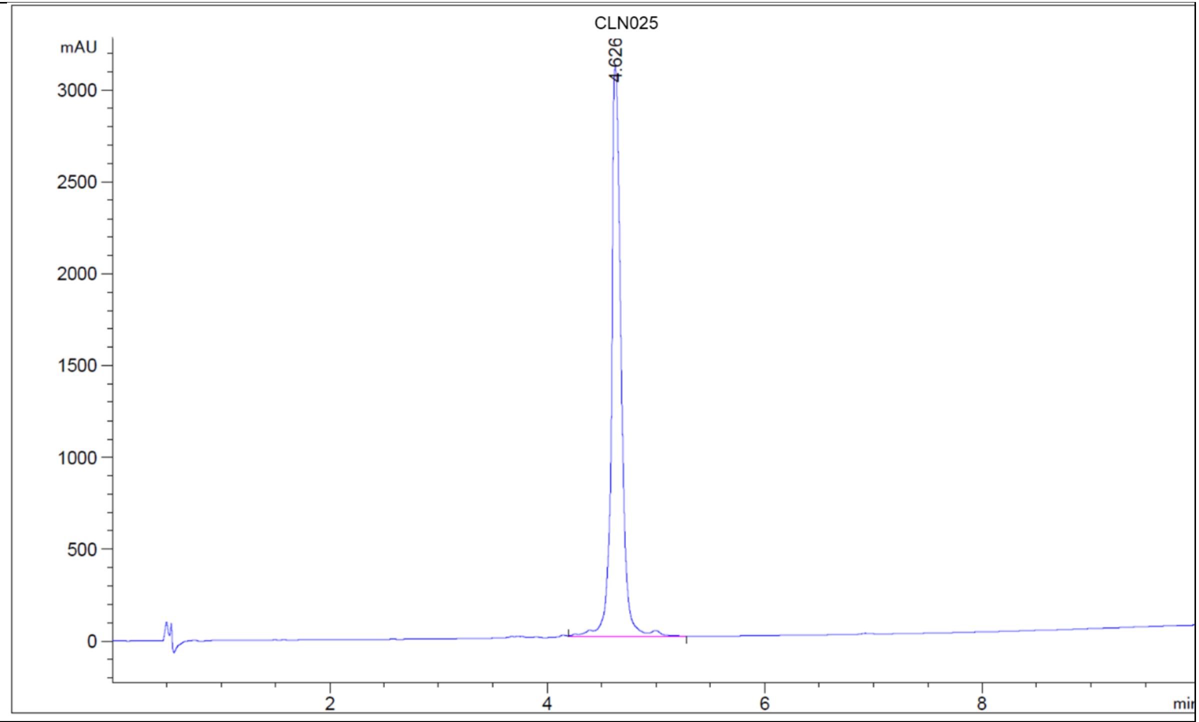

Figure S2. Analytical high-performance liquid chromatography (HPLC) of CLN025 peptide synthesis

Note S1. *Circular Dichroism*

Circular dichroism (CD) was used to validate the structure of the hairpin as it is sensitive to protein secondary structure[1]. CLN025 was prepared at 0.05 mg/mL (Figure S3(a) low) in 25 mM sodium phosphate buffer and a signal was observed which was consistent with results of Honda et al.[2] Concentration induced irreversible aggregation was also investigated by preparing CLN025 to 150

mg/mL in 25 mM sodium phosphate buffer + 250 mM NaOH and diluting to 0.05 mg/mL using 25 mM sodium phosphate buffer immediately after preparation (figure S3(a) diluted) and 24 hours after preparation (figure S3(a) diluted 24 hr). If these conditions were to cause irreversible aggregation, we would expect to see significant differences between the figure S3(a) low, diluted, and diluted 24 hr samples, however none are observed, and marginal differences in signal amplitude can be attributed to small concentration differences following the 3000x dilution.

For completeness the hairpin folded fraction was also compared to that from Honda *et al.*[2] as a function of temperature (figure S3(b)). This was done by monitoring the CD signal at 229 nm between 5 °C and 90 °C in steps of 1 °C at a heating rate of 1 °C/min with a 30 second settling time between data points. Temperature of the sample was monitored by a thermocouple submerged in the sample solution above the CD beam path. In the original work of Honda *et al.*[2], the CLN025 hairpin was prepared without the presence of capping groups on the N/C termini, however acetyl and amide groups were included in this work. The data show that the presence of the acetyl and amide caps present in the CLN025 used in this study result in a reduction in melting temperature from 69.6 °C to 57.3 °C compared with Honda *et al.*[2], however as hairpin is not fully unfolded in either study at the maximum studied temperature, the calculation of this melting temperature requires a degree of data extrapolation and are therefore difficult to compare reliably. A potential origin for the decreased thermal stability in the capped hairpin as opposed to the uncapped hairpin could be disruption of aromatic stacking between Tyr/Trp residues which takes place around the N and C termini by the amide/acetyl groups. In any case, under ambient conditions, the hairpin folded fraction is expected to be essentially identical.

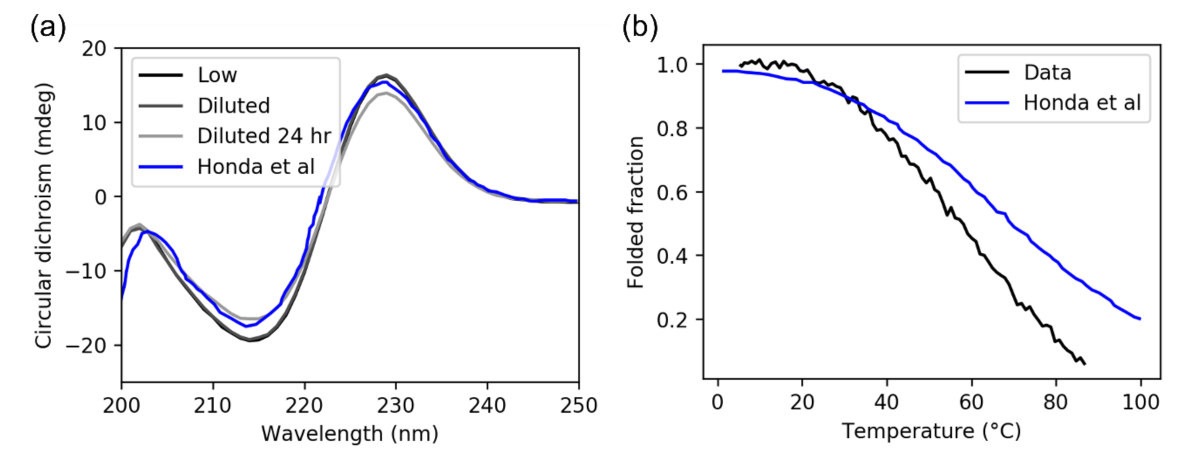

Figure S3. Circular dichroism spectra of CLN025 prepared at 0.05 mg/mL (low), diluted to 0.05 mg/mL from 150 mg/mL (diluted), diluted to 0.05 mg/mL from 150 mg/mL after incubation overnight at room temperature (diluted 24 hr), and from Honda *et al.*[2]. Honda *et al.* data scaled and vertically shifted to allow for easier comparison to account for differences in concentration and experimental setup. Data taken using WebPlotDigitizer[3] (a). Folded fraction of CLN025 as a function of temperature determined by monitoring circular dichroism at 229 nm

Table S1. Atom force field parameters according to McKiernan *et al.*[4] for CLN025. Atom named by amino acid, followed by element type, followed by an additional atom specifier according to Supplentary figure 1. Contains 79 distinct atom types for CLN025. Force field parameters for water (O_w and H_w) and Na^+ also described according to Mancinelli *et al.*[5].

Atom Type	ε (kJ/mol)	σ (Å)	Charge (e)
TyrN, ThrN, GlyN, TrpN	0.7112800	3.249998524	-0.4157
AspN, GluN	0.7112800	3.249998524	-0.5163

ProN	0.7112800	3.249998524	-0.2548
NheN	0.7112800	3.249998524	-0.4630
TrpNE1	0.7112800	3.249998524	-0.3418
TyrO, ThrO, GlyO, TrpO, AceO	0.8786400	2.959921901	-0.5679
AspO, GluO	0.8786400	2.959921901	-0.5819
ProO	0.8786400	2.959921901	-0.5748
GluOE	0.8786400	2.959921901	-0.8188
AspOD	0.8786400	2.959921901	-0.8014
ThrOG	0.8803136	3.066473388	-0.6761
TyrOH	0.8803136	3.066473388	-0.5579
TyrHA	0.0656888	2.471353044	0.0876
AspHA	0.0656888	2.471353044	0.0880
GluHA	0.0656888	2.471353044	0.1105
ProHA	0.0656888	2.471353044	0.0641
ThrHA	0.0656888	2.471353044	0.1007
GlyHA	0.0656888	2.471353044	0.0698
TrpHA	0.0656888	2.471353044	0.1123
TyrH, ThrH, GlyH, TrpH	0.0656888	1.069078462	0.2719
AspH, GluH	0.0656888	1.069078462	0.2936
NheH	0.0656888	1.069078462	0.2315
TrpHE1	0.0656888	1.069078462	0.3412
TyrHB	0.0656888	2.649532788	0.0295
AspHB	0.0656888	2.649532788	-0.0122
GluHB	0.0656888	2.649532788	-0.0173
GluHG	0.0656888	2.649532788	-0.0425
ProHD	0.0656888	2.471353044	0.0391
ProHG	0.0656888	2.649532788	0.0213
ProHB	0.0656888	2.649532788	0.0253
ThrHB	0.0656888	2.471353044	0.0043
TrpHB	0.0656888	2.649532788	0.0339
AceHH3	0.0656888	2.649532788	0.1123
ThrHG2	0.0656888	2.649532788	0.0642
TyrHH	0.0000000	0.000000000	0.3992
ThrHG1	0.0000000	0.000000000	0.4102
TyrHD	0.0627600	2.599642460	0.1699
TyrHE	0.0627600	2.599642460	0.1656
TrpHD1	0.0627600	2.510552588	0.2062
TrpHZ2	0.0627600	2.599642460	0.1572
TrpHH2	0.0627600	2.599642460	0.1417
TrpHZ3	0.0627600	2.599642460	0.1447
TrpHE3	0.0627600	2.599642460	0.1700
TyrCA	0.4577296	3.399669508	-0.0014
AspCA	0.4577296	3.399669508	0.0381
GluCA	0.4577296	3.399669508	0.0397
ProCA	0.4577296	3.399669508	-0.0266
ThrCA	0.4577296	3.399669508	-0.0389
GlyCA	0.4577296	3.399669508	-0.0252
TrpCA	0.4577296	3.399669508	-0.0275
TyrC, ThrC, GlyC, TrpC	0.3598240	3.399669508	0.5973
AspC, GluC	0.3598240	3.399669508	0.5366

ProC	0.3598240	3.399669508	0.5896
AceC	0.3598240	3.399669508	0.5972
TyrCB	0.4577296	3.399669508	-0.0152
AspCB	0.4577296	3.399669508	-0.0303
GluCB	0.4577296	3.399669508	0.0560
GluCG	0.4577296	3.399669508	0.0136
ProCD	0.4577296	3.399669508	0.0192
ProCG	0.4577296	3.399669508	0.0189
PRoCB	0.4577296	3.399669508	-0.0070
ThrCB	0.4577296	3.399669508	0.3654
TrpCB	0.4577296	3.399669508	-0.0050
AceCH3	0.4577296	3.399669508	-0.3662
ThrCG	0.4577296	3.399669508	-0.2438
TyrCZ	0.3598240	3.399669508	0.3226
AspCG	0.3598240	3.399669508	0.7994
GluCD	0.3598240	3.399669508	0.8054
TyrCG	0.3598240	3.399669508	-0.0011
TyrCD	0.3598240	3.399669508	-0.1906
TyrCE	0.3598240	3.399669508	-0.2341
TrpCG	0.3598240	3.399669508	-0.1415
TrpCD1	0.3598240	3.399669508	-0.1638
TrpCE2	0.3598240	3.399669508	0.1380
TrpCD2	0.3598240	3.399669508	0.1243
TRpCZ2	0.3598240	3.399669508	-0.2601
TrpCZ3	0.3598240	3.399669508	-0.1972
TrpCE3	0.3598240	3.399669508	-0.2387
TrpCH2	0.3598240	3.399669508	-0.1134
O_w	0.6500000	3.166000000	-0.8476
H_w	0.0000000	0.000000000	0.4238
Na^+	0.5144000	2.299000000	1.0000

Note S2. *Reducing atom types*

The reference potential for water was the SPC/E model,[6] the Na^+ force field was taken from Mancinelli[7] and the CLN025 force field taken AMBER-FB15 potential from McKiernan *et al.*[4] This potential describes CLN025 using 79 distinct atom types (characterised through the two Lennard-Jones (LJ) and charge, see supplementary tables S1 and S2 and supplementary figure S5 for atom naming conventions and parameters). As the number of distinct interatomic correlations for J distinct atom types is equal to $J(J+1)/2$[8], to improve simulation efficiency atoms with identical LJ parameters and highly similar charges were described by a single atom type of charge weighted by the occurrence within the molecule to leave the total charge unaltered. This procedure reduces 79 distinct atom types to 33. The raw and modified force field can be found in supplementary tables S1 and S2.

Table S2. Atom force field parameters used in EPSR for CLN025, water, and Na^+, contains 36 distinct atom types

N1 (TryN, ThrN, GlyN, TrpN)	0.711280	3.249999	-0.41570
N2 (AspN, GluN)	0.711280	3.249999	-0.51630
N3 (ProN)	0.711280	3.249999	-0.25480
N4 (NheN)	0.711280	3.249999	-0.46300
N5 (TrpNE1)	0.711280	3.249999	-0.34180

O1 (TryO, AspO, GluO, ProO, ThrO, GlyO, TrpO, AceO)	0.878640	2.959922	-0.57107
O2 (GluOE, AspOD)	0.878640	2.959922	-0.81010
O3 (ThrOG, TyrOH)	0.880314	3.066473	-0.60518
H1 (TyrHA, AspHA)	0.065689	2.471353	0.08770
H2 (GlyHA, ProHA)	0.065689	2.471353	0.06790
H3 (GluHA, ThrHA, TrpHA)	0.065689	2.471353	0.10605
H4 (TyrH, AspH, GluH, ThrH, GlyH, TrpH) (exchangable)	0.065689	1.069078	0.27672
H5 (NheH) (exchangable)	0.065689	1.069078	0.23150
H6 (TrpHE1) (exchangable)	0.065689	1.069078	0.34120
H7 (TyrHB, AspHB, GluHB, GluHG, ProHD, ProHG, ProHB, ThrHB, TrpHB)	0.065689	2.609937	0.01276
H8 (AceHH3)	0.065689	2.649533	0.11230
H9 (ThrHG2)	0.065689	2.649533	0.06420
H10 (TyrHH, ThrHG1) (exchangable)	0.000000	0.000000	0.40360
H11 (TyrHD, TyrHE, TrpHE3)	0.062760	2.599642	0.16792
H12 (TrpHD1)	0.062760	2.510553	0.20620
H13 (TrpHZ2, TrpHH2, TrpHZ3)	0.062760	2.599642	0.14787
C1 (TyrCA, AspCA, GluCA, ProCA, ThrCA, GlyCA, TrpCA, TyrCB, AspCB, GluCB, GluCG, ProCD, ProCG, ProCB, TrpCB)	0.457730	3.399670	-0.00319
C2 (TyrC, AspC, GluC, ProC, ThrC, GlyC, TrpC, AceC)	0.359824	3.399670	0.58556
C3 (ThrCB)	0.457730	3.399670	0.36540
C4 (AceCH3)	0.457730	3.399670	-0.36620
C5 (ThrCG)	0.457730	3.399670	-0.24380
C6 (TyrCZ)	0.359824	3.399670	0.32260
C7 (AspCG, GluCD)	0.359824	3.399670	0.80240
C8 (TyrCG)	0.359824	3.399670	-0.00110
C9 (TyrCD, TrpCD1, TrpCZ3)	0.359824	3.399670	-0.18808
C10 (TrpCE2, TrpCD2)	0.359824	3.399670	0.13115
C11 (TrpCG, TrpCH2)	0.359824	3.399670	-0.12745
C12 (TyrCE, TrpCZ2, TrpCE3)	0.359824	3.399670	-0.23793

O_w	0.650000	3.1660	-0.84760
H_w (exchangeable)	0.000000	0	0.42380
Na^+	0.514400	2.2990	1.00000

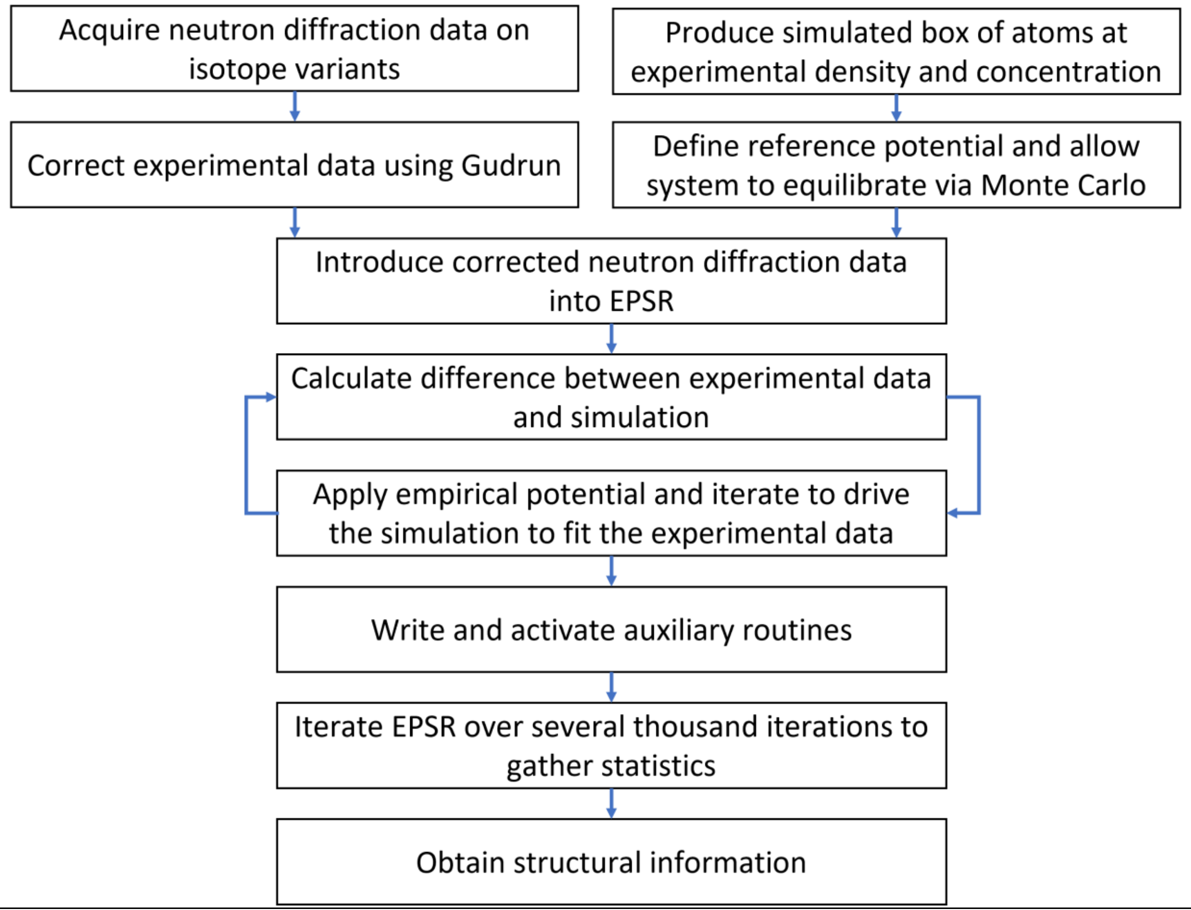

Figure S4. Workflow of empirical potential structure refinement. Figure adapted from[9]. The quality of fit between the simulated and experimental scattering data is quantified through the R-factor, defined by the following equation, where $D_i(Q)$ and $F_i(Q)$ are the experimental and simulated scattering data, M is the number of datasets provided by isotopic substitution, and $n_Q(i)$ is the number of Q values in the ith dataset:

$$R = \frac{1}{M}\sum_i \frac{1}{n_Q(i)}\sum_Q [D_i(Q) - F_i(Q)]^2$$

A perfect agreement is therefore described by $R = 0$.

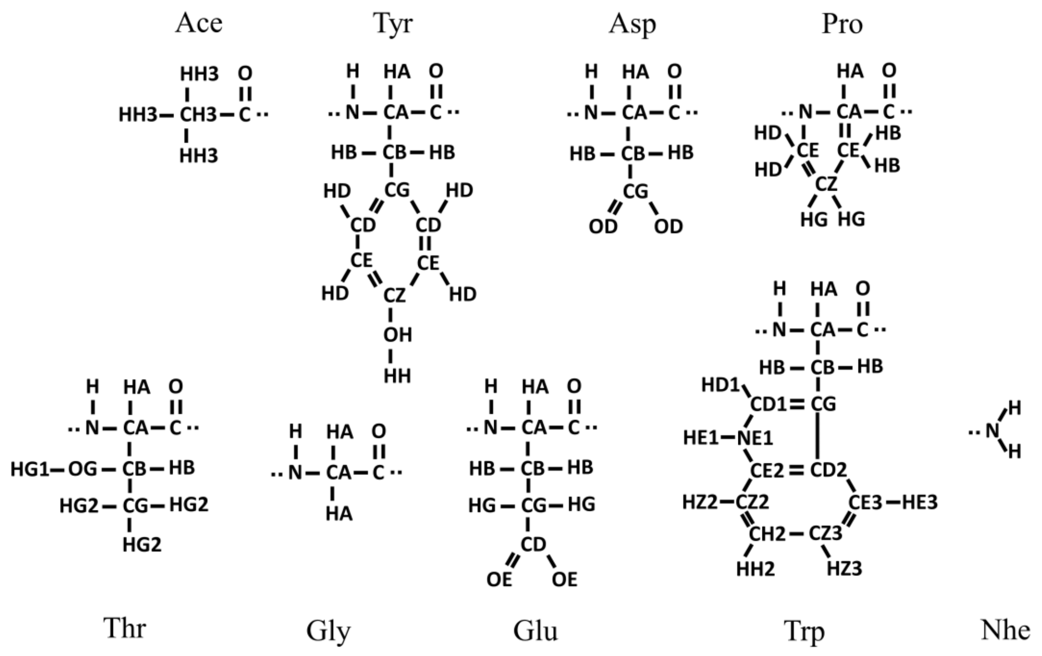

Figure S5. Atom naming convention for amino acids and capping groups present in CLN025 hairpin according to the AMBER-FB15 forcefield published by McKiernan et al[4].

Note S3. *Low Q data fitting*

Equation 5 in the main manuscript was used to fit the stitched neutron scattering data over a wide Q-range i.e. 0.003 $Å^{-1}$ to 3 $Å^{-1.}$

In order to validate this fit, the unstitched Zoom and NIMROD data sets were also fit individually with equations S1 and S2 respectively.

Eqn S1:

$$I(Q) = \varphi \Delta\rho^2 V_{block} P_{EC}(Q) S_{ER}(Q) + \gamma \cdot Q^{-n} + bkg$$

Eqn S2:

$$I(Q) = \varphi \Delta\rho^2 V_{block} P_{EC}(Q) S_{ER}(Q) + \alpha \cdot e^{-\frac{(Q-Q_{ip})^2}{2\sigma_{ip}^2}} + \beta \cdot e^{-\frac{(Q-Q_{is})^2}{2\sigma_{is}^2}} + bkg$$

Figure S6 shows these fits to both of these data sets and demonstrates that the unstitched data is adequately fit by individual components of equation 5.

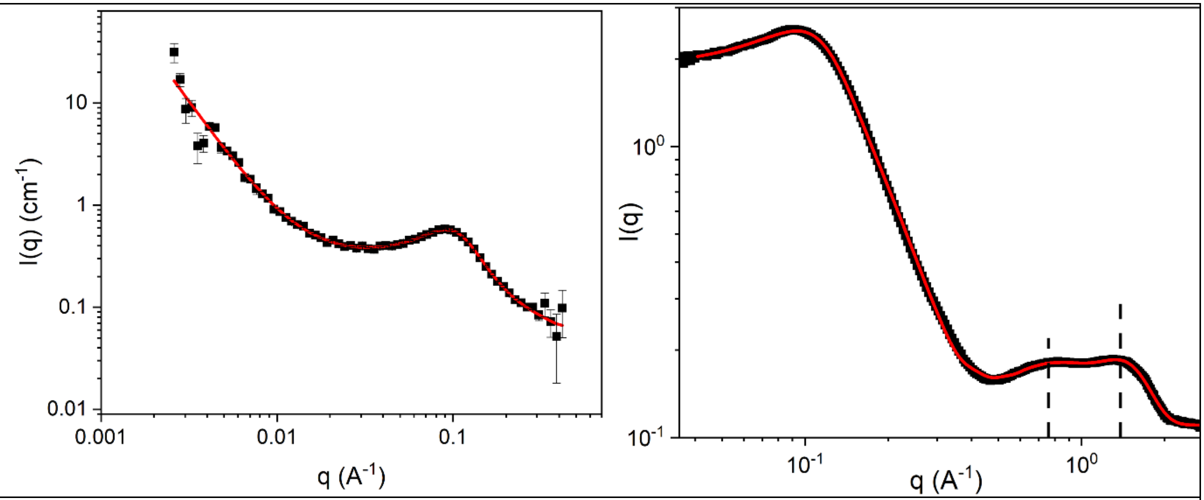

Figure S6. The unstitched (left) Zoom and (right) NIMROD data sets. The red lines denote the fits of equations (left) Eqn S1 and (right) Eqn S2.

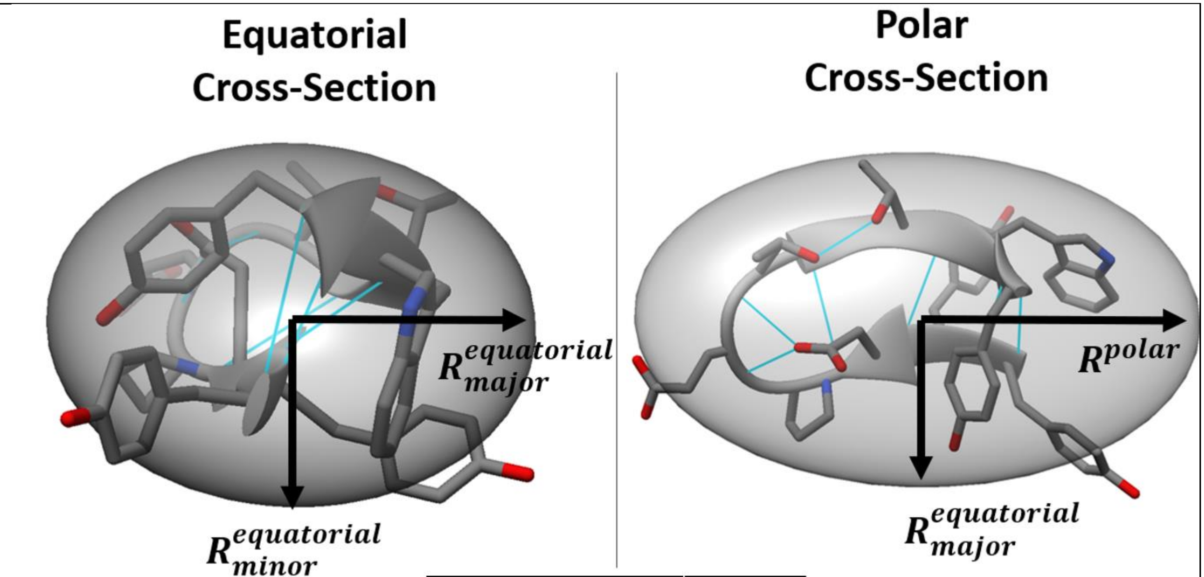

Parameter	Value (Å)
$R_{major}^{equatorial}$	6.6
$R_{minor}^{equatorial}$	4.8
R^{Polar}	11

Figure S7. Elliptical cross sections of CLN025 hairpin used to fit the low Q data presented in main text figure 3. Figure shows the (left) elliptical and (right) polar cross-sections of the NMR structure of CLN025 (PDB code: 2RVD) The structure was fitted with an ellipsoid envelope using USCF Chimera's measure inertia function.[16] The extracted radii of this fit are given in the table (bottom).

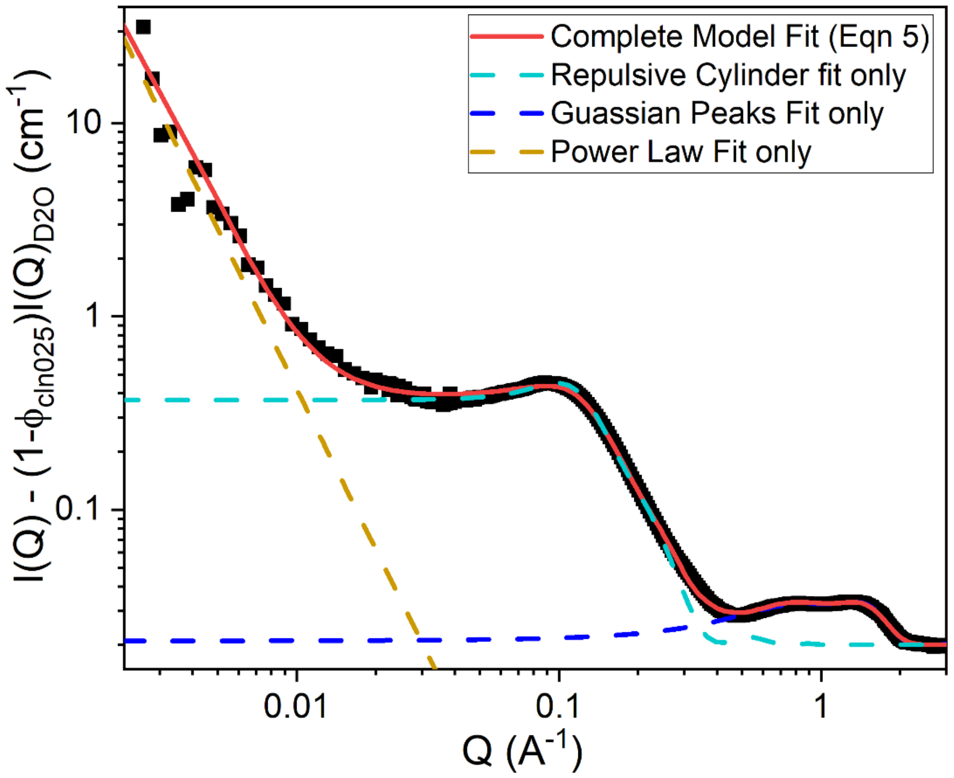

Figure S8. Stitched SANS/WANS curve of CLN025 at 150mg/ml suspended in a solution of 250mM NaOH/100% D_2O. The solid red line shows the fit to the data of our empirical SANS model shown in equation 5 (Chi-squared: 4). The dashed lines all show the fits of individual terms of the model. The dark blue dashed line (fitted between Q = 0.4 $Å^{-1}$ and Q = 3 $Å^{-1}$) shows the fit for the two gaussian peak terms in equation 5 (Chi-squared: $3x10^{-7}$). The cyan dashed line (fitted between Q = 0.03 $Å^{-1}$ and Q = 0.4 $Å^{-1}$) shows the fit for the repulsive cylinder term in equation 5 (Chi-squared: 0.006). The dark yellow line (fitted between Q = 0.002 $Å^{-1}$ and Q = 0.006 $Å^{-1}$) shows the power law term from equation 5 (Chi-squared: 3.7). All dashed lines have been extended to full axis range to show how each term behaves at various Q-ranges.

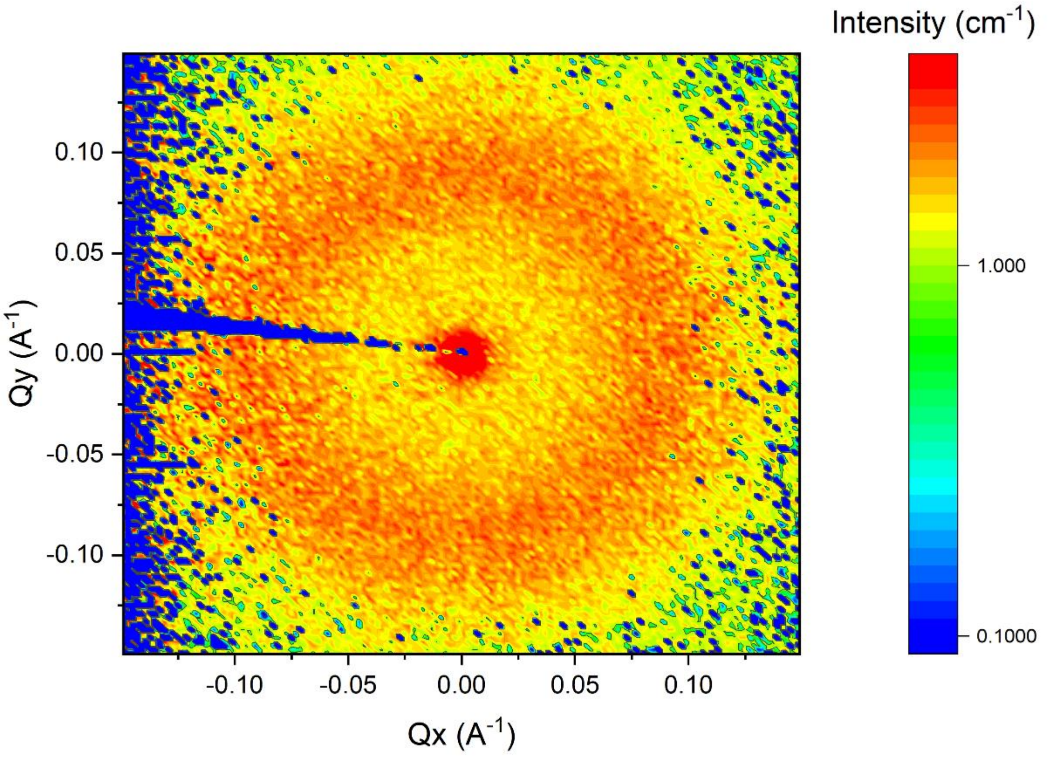

Figure S9: Colour fill map of the 2D scattering pattern of CLN025 at 150mg/ml. The pattern shows a circular ring which corresponds the 'peak' in the 1D scattering pattern at $Q \approx 0.1$ Å^{-1} (Figure 2a). The symmetry of the ring (i.e. the ring is circular not elliptical) suggests that on the length-scale of the CLN025 'stacks' (approx. 60 Å) there is no anisotropy or preferential direction i.e. the 'stacks' are randomly orientated. Note there is a radial line of no scattering (from centre to $Q_y \approx 0.025$ Å^{-1}), caused by the beamstop arm holding the beamstop at the centre of the detector.

Note S4 *CLN025 self assembly*

The SANS data suggests a stacked hairpin structure involving 7 CLN025 monomers, however it is incapable of providing the structure of a stack with atomic resolution. To achieve this we built a stack of seven all atom hairpin monomers using a custom built molecular docking Monte Carlo simulation, as detailed in supplementary information note S2 and figure S10. This held a host "flat" CLN025 molecule at a fixed position (COM at 0,0,0) and a second docking flat CLN025 molecule 8.5 Å above it in the z direction that had been rotated by π radians around the z axis. The flat conformation of CLN025 molecule was generated by allowing the molecule to rotate about its centre of mass to minimize the absolute value of the z coordinate on each atom, and the π rotation on docking molecule done as it was demonstrated that two similarly aligned molecules result in highly unfavourable interactions due to steric interactions and proximity of the negatively charged Glu5 side chains. This does not allow a stack to build as suggested by the SANS data.

The docking molecule was moved in the xy plane between -8.0 and 8.0 in the x direction and -5.0 and 5.0 in the y direction in increments of 0.5 Å. At each point docking molecule was allowed to perform 100 Monte Carlo iterations. Each iteration consisted of a randomly chosen positive or negative rotation in the alpha, beta, or gamma direction as defined by the Euler angles by $\pi/280$ radians, and the total interaction energy between the two hairpins was calculated after each iteration according to the reference potential[4]. The results of this method of establishing the most favourable location for

molecular docking are shown in figures S10-15. The xy coordinate with the lowest potential energy that also allowed for a stack to be built that was consistent with the elliptical cross section predicted by the SANS data was then selected (see SI for full maps), and further equilibrated for 1000 Monte Carlo iterations to optimise the dimer. This procedure was repeated iteratively using the docking molecule as the new host molecule until a stack of 7 was built.

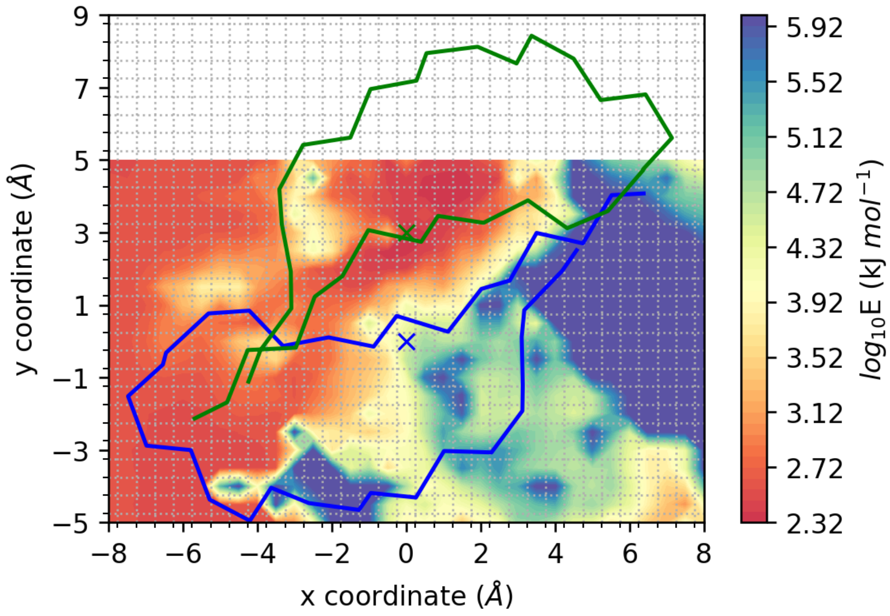

Figure S10. Energy landscape for molecular docking between host CLN025 hairpin at position 0 and docking CLN025 hairpin at position 1. Hairpins are displayed in a simplified manner showing only the hairpin backbone (host = blue, docking = green). Final optimised relative positions are shown for host and docking molecule with corresponding centre of mass coordinates of the two hairpins shown as coloured x's. White areas above y>5 indicate areas where hairpin-hairpin interaction energies were not calculated, but are included to help the reader visualize the relative positions of the two hairpins.

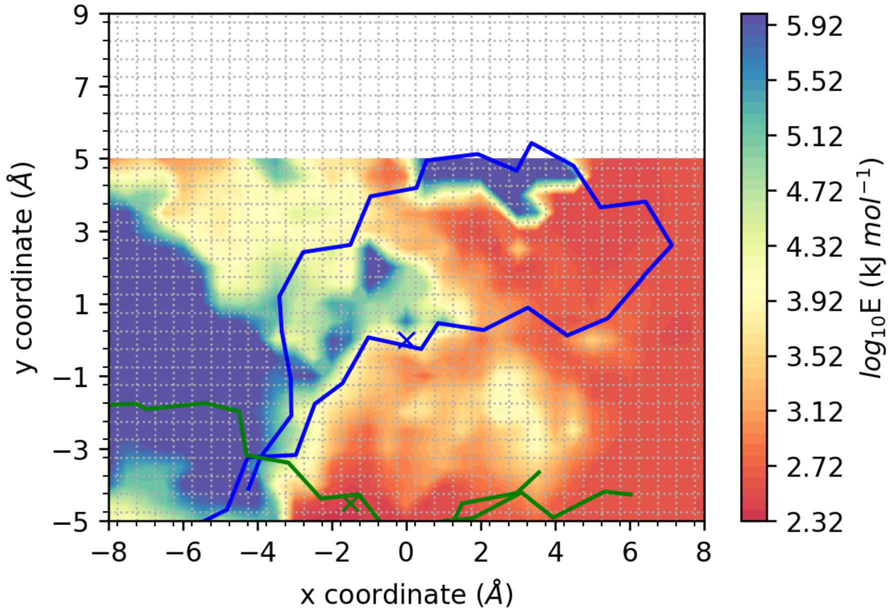

Figure S11. Energy landscape for molecular docking between host CLN025 hairpin at position 1 and docking CLN025 hairpin at position 2. Hairpins are displayed in a simplified manner showing

only the hairpin backbone (host = blue, docking = green). Final optimised relative positions are shown for host and docking molecule with corresponding centre of mass coordinates of the two hairpins shown as coloured x's. White areas above y>5 indicate areas where hairpin-hairpin interaction energies were not calculated, but are included to help the reader visualize the relative positions of the two hairpins.

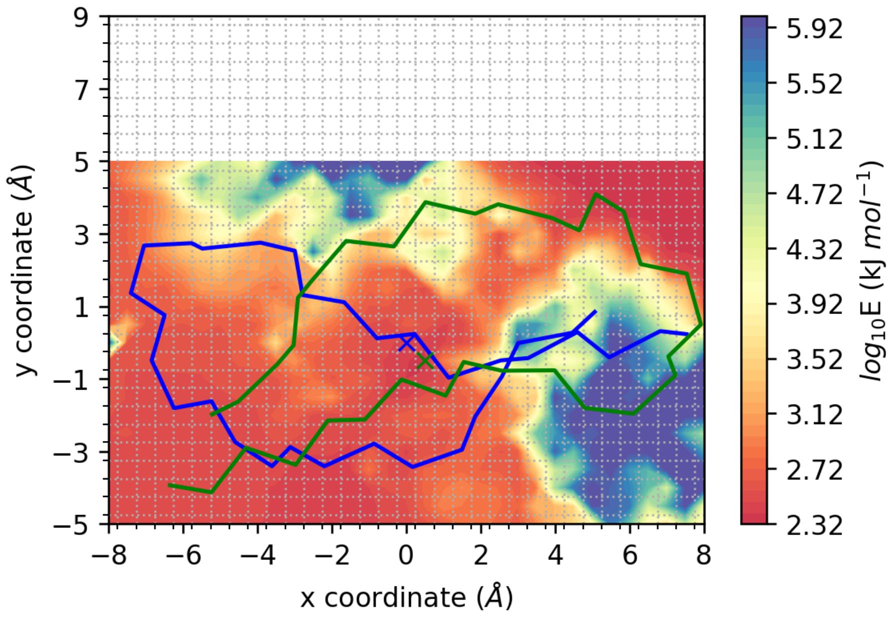

Figure S12. Energy landscape for molecular docking between host CLN025 hairpin at position 2 and docking CLN025 hairpin at position 3. Hairpins are displayed in a simplified manner showing only the hairpin backbone (host = blue, docking = green). Final optimised relative positions are shown for host and docking molecule with corresponding centre of mass coordinates of the two hairpins shown as coloured x's. White areas above y>5 indicate areas where hairpin-hairpin interaction energies were not calculated, but are included to help the reader visualize the relative positions of the two hairpins.

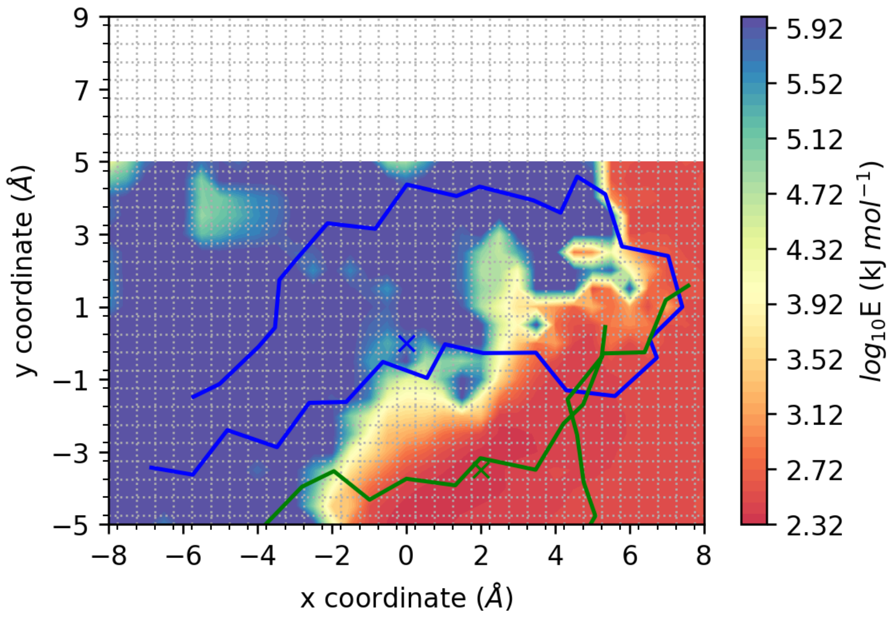

Figure S13. Energy landscape for molecular docking between host CLN025 hairpin at position 3 and docking CLN025 hairpin at position 4. Hairpins are displayed in a simplified manner showing only the hairpin backbone (host = blue, docking = green). Final optimised relative positions are shown for host and docking molecule with corresponding centre of mass coordinates of the two hairpins shown as coloured x's. White areas above y>5 indicate areas where hairpin-hairpin interaction energies were not calculated, but are included to help the reader visualize the relative positions of the two hairpins.

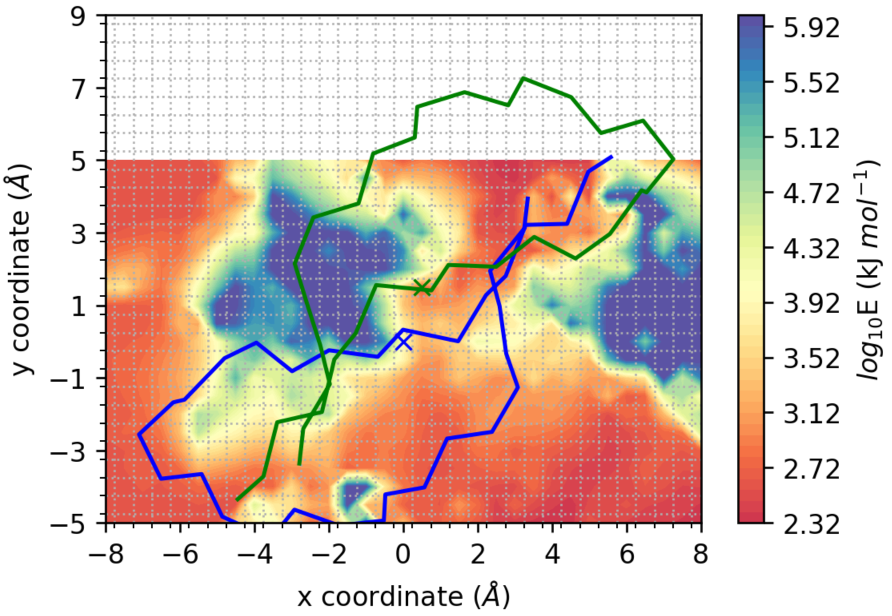

Figure S14. Energy landscape for molecular docking between host CLN025 hairpin at position 4 and docking CLN025 hairpin at position 5. Hairpins are displayed in a simplified manner showing only the hairpin backbone (host = blue, docking = green). Final optimised relative positions are shown for host and docking molecule with corresponding centre of mass coordinates of the two hairpins shown as coloured x's. White areas above y>5 indicate areas where hairpin-hairpin interaction energies were not calculated, but are included to help the reader visualize the relative positions of the two hairpins.

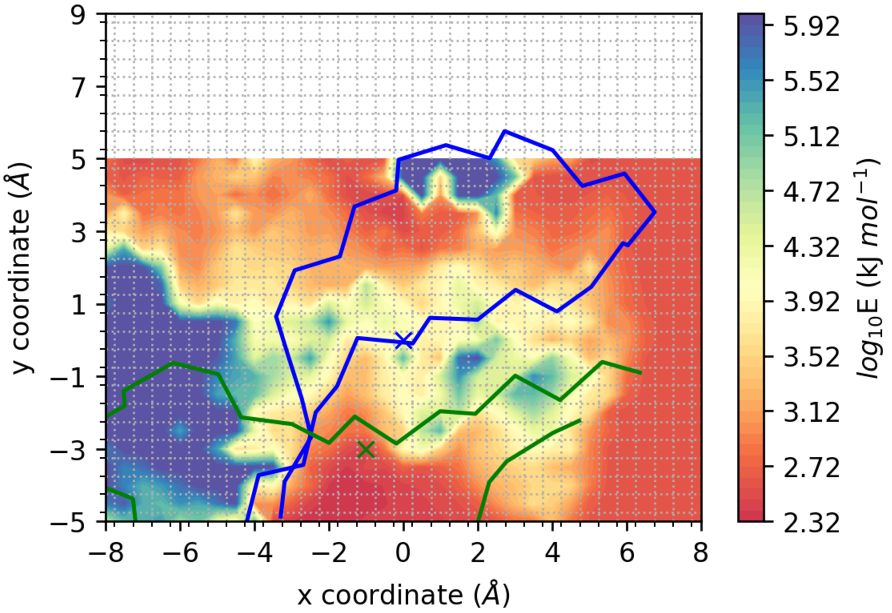

Figure S15. Energy landscape for molecular docking between host CLN025 hairpin at position 5 and docking CLN025 hairpin at position 6. Hairpins are displayed in a simplified manner showing only the hairpin backbone (host = blue, docking = green). Final optimised relative positions are shown for host and docking molecule with corresponding centre of mass coordinates of the two hairpins shown as coloured x's. White areas above y>5 indicate areas where hairpin-hairpin interaction energies were not calculated, but are included to help the reader visualize the relative positions of the two hairpins.

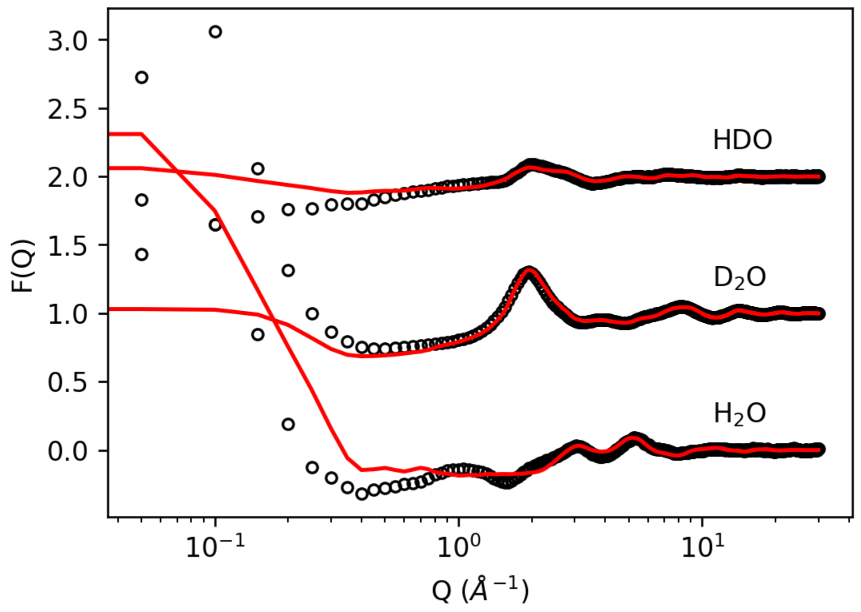

Figure S16. The differential scattering cross section (I(Q)) obtained from the neutron diffraction data on the NIMROD instrument (black circles) for the CLN025 hairpin in H_2O, D_2O and HDO at 298 K and the EPSR obtained data (red line). Units of the differential cross sections are barns/atom/sr (1 barn = 10^{-28} m^2).

Note S5. *Hydrophobicity analysis*

Mapping the surface in this way (see figure 4 in main text) using the proposed Δ parameter therefore gives us a measure of hydrophobicity, where strongly positive or negative Δ values, corresponding to hydrogen bond acceptors and donors respectively, and are hydrophilic, while values close to 0 are hydrophobic. We can therefore consider the absolute value of the hydrogen bonding index Δ to be defined as the hydrophobicity index $|\Delta|$. For completeness it is interesting to consider how the calculated measure of hydrophobicity we propose here compares with other available hydrophobicity indexes. To do this we consider 5 previous studies which attempt to quantify amino acid hydrophobicity[10–14], each of which assigns a numerical value to each of the 20 naturally occurring amino acids. We normalise these scales to vary between 0 – 1 with 0 being hydrophobic and 1 being hydrophilic, such that they are equivalent to the hydrophobicity index $|\Delta|$. We therefore compare the two by calculating the average $|\Delta|$ value for each amino acid on CLN025. This is done by calculating the average $|\Delta|$ value for each atom belonging to each residue weighted by how much solvent accessible service (SAS) each atom contributes to the whole residues total SAS. The value for multiply occurring amino acids (Tyr and Thr) calculated by taking the arithmetic mean of each occurrence. The results are reported in figure S17.

These results show reasonable agreement between $|\Delta|_{\Delta}$ and $|\Delta|_{Lit}$ for all amino acids except Trp and Tyr, however in all cases the method proposed here predicts greater hydrophilic character than literature values. This is likely because calculations are solely based on atoms that are solvent exposed, which by definition are likely to be more hydrophilic and result in higher calculated $|\Delta|$. In the example of Tyr, the hydrophilic side chain hydroxyl group accounts for ~50% of the total SAS in CLN025, which is far higher than the 14.5% this group contributes to the total SAS in monomeric Tyr[15]. In the case of Trp the total hydrophilic SAS arising from the indole NH and the solvent exposed backbone amine and carbonyl group account for 30.4% of the total SAS. Furthermore, the hydrophobic six membered carbon ring on the indole side chain sits directly between the hydrophilic and well solvent exposed Tyr2 OH hydrogen (donor site) and the acetyl cap backbone oxygen (acceptor site), meaning it gains a more hydrophilic character than it otherwise would.

This observation indicates that hydrophobicity of an amino acid is strongly context dependent. This point is exemplified further if we consider the two occurrences of Thr in CLN025. In both instances the hydrophilic side chain hydroxyl group is mostly buried, with the SAS of the hydrogen on this group being 0.97 $Å^2$ and 2.55 $Å^2$ for Thr6 and Thr8 respectively. However, in Thr6 the backbone oxygen is strongly solvent exposed, with an SAS of 26.87 $Å^2$. This strongly exposed hydrogen bond acceptor site results in $|\Delta| = 0.72$ for Thr6, but only $|\Delta| = 0.48$ for Thr8, hence Thr6 is much more hydrophilic.

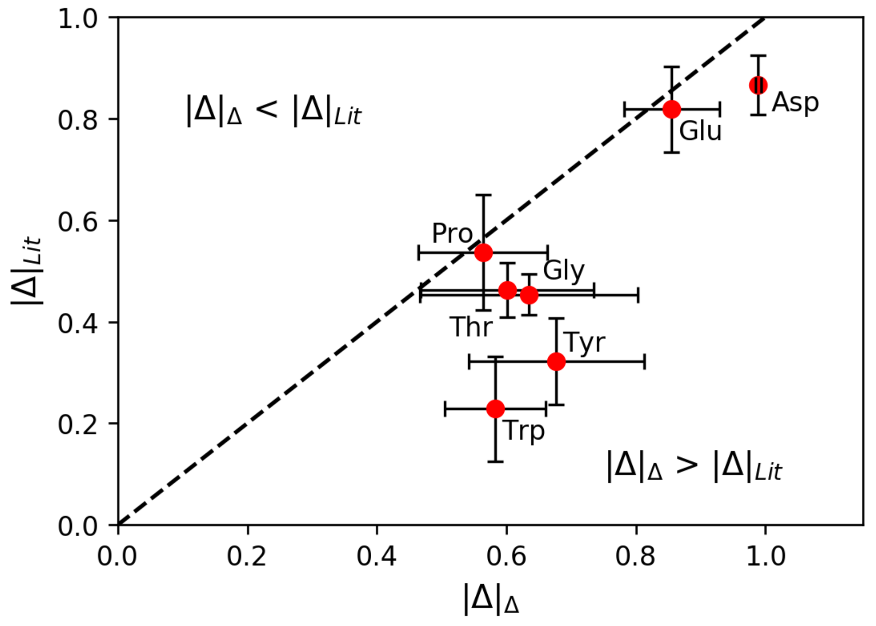

Figure S17. Comparing the hydrophobicity $|\Delta|$ calculated in this work $|\Delta|_\Delta$ to literature values $|\Delta|_{Lit}$[10–14]. Dashed black line where $|\Delta|_\Delta = |\Delta|_{Lit}$. Vertical error bars correspond to the standard error across the five considered literature hydrophobicity indices. Horizontal error bars correspond to standard error for calculated $|\Delta|_\Delta$ values.

References

1. Kelly, S. M., Jess, T. J. & Price, N. C. How to study proteins by circular dichroism. *Biochim. Biophys. Acta - Proteins Proteomics* **1751**, 119–139 (2005).
2. Honda, S. *et al.* Crystal structure of a ten-amino acid protein. *J. Am. Chem. Soc.* **130**, 15327–15331 (2008).
3. Rohatgi, A. WebPlotDigitizer. (2022). Available at: https://automeris.io/WebPlotDigitizer. (Accessed: 2nd February 2023)
4. McKiernan, K. A., Husic, B. E. & Pande, V. S. Modeling the mechanism of CLN025 beta-hairpin formation. *J. Chem. Phys.* **147**, 104107 (2017).
5. Mancinelli, R., Botti, A., Bruni, F., Ricci, M. A. & Soper, A. K. Hydration of sodium, potassium, and chloride ions in solution and the concept of structure maker/breaker. *J. Phys. Chem. B* **111**, 13570–13577 (2007).
6. Mark, P. & Nilsson, L. Structure and dynamics of the TIP3P, SPC, and SPC/E water models at 298 K. *J. Phys. Chem. A* **105**, 9954–9960 (2001).
7. Mancinelli, R., Botti, A., Bruni, F., Ricci, M. A. & Soper, A. K. Perturbation of water structure due to monovalent ions in solution. *Phys. Chem. Chem. Phys.* **9**, 2959–2967 (2007).
8. Soper, A. K. *et al. Empirical Potential Structure Refinement A User's Guide*. (2017).
9. Laurent, H. The Biological Role of Water in Extreme Conditions. (University of Leeds, 2021).
10. Kyte, J. & Doolittle, R. F. A simple method for displaying the hydropathic character of a protein. *J. Mol. Biol.* **157**, 105–132 (1982).
11. Wimley, W. C. & White, S. H. Experimentally determined hydrophobicity scale for proteins at membrane interfaces. *Nat. Struct. Mol. Biol.* **3**, 842–848 (1996).

12. Hessa, T. *et al.* Recognition of transmembrane helices by the endoplasmic reticulum translocon. *Nature* **433**, 377–381 (2005).

13. Moon, C. P. & Fleming, K. G. Side-chain hydrophobicity scale derived from transmembrane protein folding into lipid bilayers. *Proc. Natl. Acad. Sci.* **108**, 10174–10177 (2011).

14. Zhao, G. & London, E. An amino acid “transmembrane tendency” scale that approaches the theoretical limit to accuracy for prediction of transmembrane helices: Relationship to biological hydrophobicity. *Protein Sci.* **15**, 1987–2001 (2006).

15. Mostad, A., Nissen, H. M. & Romming, C. Crystal Structure of L-Tyrosine. *Acta Chem. Scaninavica* **26**, 3819–3833 (1972).

16. Pettersen EF, Goddard TD, Huang CC, Couch GS, Greenblatt DM, Meng EC, Ferrin TE., UCSF Chmiera - a visualisation system for exploratory research and analysis, *J Comput Chem*. **25**, 1605-12 (2004).
